# Cyclodextrins: promising scaffolds for MRI contrast agents

**DOI:** 10.1039/d1ra04084g

**Published:** 2021-09-17

**Authors:** Berthe Sandra Sembo-Backonly, François Estour, Géraldine Gouhier

**Affiliations:** Normandie Université, COBRA UMR 6014, FR 3038, INSA Rouen, CNRS, IRCOF 1 Rue Tesnière 76821 Mont-Saint-Aignan France geraldine.gouhier@univ-rouen.fr

## Abstract

Magnetic resonance imaging (MRI) is a powerful tool for non-invasive, high-resolution three-dimensional medical imaging of anatomical structures such as organs and tissues. The use of contrast agents based on gadolinium chelates started in 1988 to improve the quality of the image, since researchers and industry focused their attention on the development of more efficient and stable structures. This review is about the state of the art of MRI contrast agents based on cyclodextrin scaffolds. Chemical engineering strategies are herein reported including host–guest inclusion complexation and covalent linkages. It also offers descriptions of the MRI properties and *in vitro* and *in vivo* biomedical applications of these emerging macrostructures. It highlights that these supramolecular associations can improve the image contrast, the sensitivity, and the efficiency of MRI diagnosis by targeting cancer tumors and other diseases with success proving the great potential of this natural macrocycle.

## Abstract 2

This review highlights that supramolecular associations using host–guest inclusion complexation and covalent linkages can improve the image contrast, the sensitivity, and the efficiency of MRI diagnosis.

## Graphical abstract

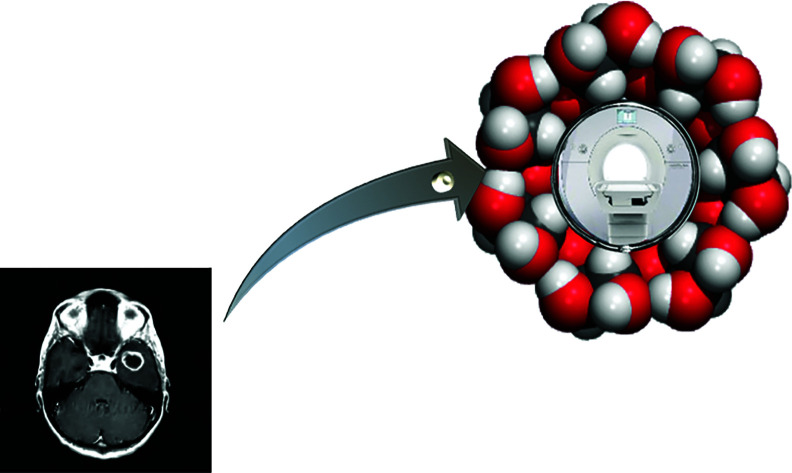

## 1. Introduction

**Fig. 1:**
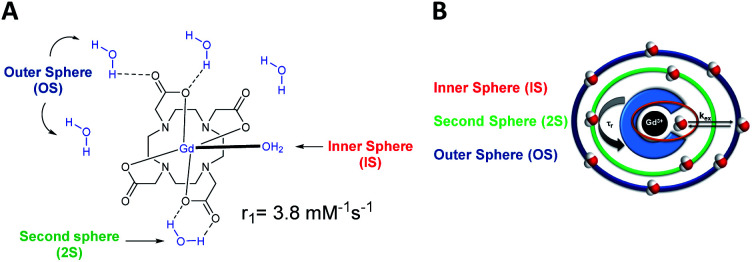
(A) Structure of the Gd-DOTA and relaxivity. (B) Schematic representation of a gadolinium chelate, its different hydration spheres and the main parameters that influence its effectiveness.

Magnetic resonance imaging (MRI) is a powerful tool for non-invasive, high-resolution three-dimensional medical imaging of anatomical structures such as organs and tissues. MRI is one of the most important imaging methods used for diagnosis and medical research because of its very high spatial and temporal resolution, excellent depth profiling capabilities, and absence of ionizing radiation in contrast with X-rays and CT scans. Despite the advantages of MRI, this technique suffers from low intrinsic sensitivity due to low natural contrast. The first contrast agent appeared in 1988 as a linear gadolinium(iii) complex DTPA(Gd) and was developed and marketed by the Bayer Schering laboratory as Magnevist®. Since the contrast agents (CAs) have become an essential diagnostic tool and are used in 35% of clinical MRI scans due to their high paramagnetism and excellent relaxation enhancement.^1^ Their injections increase the sensitivity and give more accurate images in particular between healthy and diseased tissues. Thus, a large variety of contrast agents based on linear and cyclic Gd(iii) chelates were commercialized. MRI exploits the resonance magnetic properties of water proton in the human body after short pulses of radiofrequency waves. The period of the return to equilibrium (relaxation) of hydrogen atoms after their excitation (resonance) results in a relaxation time. MRI image quality is also related to the magnetic field strength, the signal-to-noise ratio, the contrast-to-noise ratio, the spatial and the temporal resolutions. Most human MRI scanners operate at a strength of 1.5 to 3 Tesla (64–128 MHz) but high fields such as 7 to 11.7 Tesla can be used in human MRI research. Gadolinium is a non-coordinated lanthanide and it is toxic under its free form Gd(Cl)_3_(H_2_O)_6_. Indeed, its ionic ray (107.8 pm) being close to the calcium ion Ca^2+^ (114.0 pm), competition can occur with this endogenous cation and can induce interactions in many biological processes causing allergic phenomena in some patients such as urticaria, angioedema, bronchospasm, hypotension, anaphylactic shock or hypocalcemia. In 2006, Grobner *et al.* first established the link between the presence of Gd^3+^ in the body and a disease called Nephrogenic Systemic Fibrosis (NSF).^2^ This disease appears mostly in people with kidney failure. FSN becomes fatal when fibrosis affects heart tissues such as the myocardium and pericardium or lungs.^3–5^ In 2017, the Pharmacovigilance and Risk Assessment Committee (PRAC) of the European Medicines Agency (EMA) prohibited the market of linear chelates, such as DTPA(Gd) derivatives. Their studies provided convincing evidence of the accumulation of gadolinium in brain tissue.^6–8^ Indeed, an MRI signal was still detected several months after the last injection. However, the cyclic octacoordinated DOTA(Gd) (1,4,7,10-tetraacetic acid-1,4,7,10-tetraazacyclododecane) marketed as DOTAREM© is the most known and used due to its high thermodynamically stability (log β = 25.3) and the kinetic inertia of the complex (pGd = 12.1) (Fig. 1). Ten million injections of CA are performed each year for MRI diagnosis.

Thus, specific radiofrequency wave pulse sequences led to T1-weighted images, which depend on the time of longitudinal relaxation of proton spins, or in T2-weighted images, which depend on the time of the transverse relaxation of the proton spins.^9–14^ T1 agents called positive contrast agents (*e.g.*, Gd chelates) produce a brighter contrast and therefore lead to an easier detection. Their use is thereby favored over the T2 negative contrast agents (*e.g.* supermagnetic iron oxide nanoparticles) which generate a darker image. We will focus the review herein on T1 Gd(iii) MRI contrast agents mostly used clinically, even if manganese alternative emerged.^15^ The efficiency of a T1 contrast agent is characterized by a value called “relaxivity” and noted *r*_1_ that reflects its ability to increase the relaxation rate of the protons of surrounding water molecules. The relaxation rate *r*_1_ is proportional to the concentration of contrast agent in solution, the relaxivity is concentration-independent and inversely proportional to the relaxation time.

A good contrast agent generates a high relaxivity value. Access to a well resolved MR image is achieved by increasing the contrast of the image and consequently by decreasing the relaxation times of the water protons (T1). According to the well established Solomon–Bloembergen–Morgan (SBM) theory, the relaxivity is governed by many parameters.^9–12^ The structure of the complex can influence many parameters such as the aqueous environment due to the presence of three hydration spheres around the metallic complex: the inner (IS), the second (2S), and the outer (OS) spheres (Fig. 1).^16–18^ The inner sphere corresponds to the water molecule in a direct interaction with the paramagnetic cation, and it is mainly involved in the relaxivity of the contrast agent. Indeed, the exchange rates between the water molecule bounded to the lanthanide and the water molecules around the complex (OS) determine the efficiency of the contrast agent. Finally, the second coordination sphere is generated by water molecules able to form hydrogen bonds with the functions of the ligand. For DOTA(Gd) contrast agent, the influence of 2S is often overlooked or included in the contribution of the inner sphere, as it is considered much weaker than the other two.

The relaxivity value depends mainly on the aqueous environment around gadolinium following the eqn (1)–(3):

1 *r*[AC] = (1/*T*_*n*_)

2 (1/*T*_*n*_) = (1/*T*_*n*_)^IS^ + (1/*T*_*n*_)^OS^ + (1/*T*_*n*_)^2S^ (*n* = 1, 2)

3 *r*_*n* (*n*=1,2)_ = *r*^IS^ + *r*^OS^ + *r*^2S^

*r*_*n*_: relaxivity longitudinal (*n* = 1) or transverse (*n* = 2), *r*^IS^_*n*_: relaxivity of the inner sphere, *r*^OS^_*n*_: relaxivity of the outer sphere, *r*^2S^_*n*_: relaxivity of the second sphere, 1/*T*_*n*_: longitudinal (*n* = 1) or transversal (*n* = 2) relaxation rate.

However, in the presence of a hydrophilic macromolecule that generates many hydrogen bonds, this second coordination sphere gains importance and is no negligible anymore. Usually, octacoordinated ligands are used, with one water molecule in the inner hydration sphere of Gd(iii) as with DOTA(Gd), and the hydration number *q* is equal to 1.^16–18^ The presence of this water molecule is indispensable for the detection of longitudinal relaxation time T1. A higher number of water molecules can increase the *q* value to 2 or 3 but the lower kinetic and thermodynamic stabilities of the complex enhance the risk of toxic effects.^2–6^ This degree of hydration *q* is assessed by luminescence life measurement of europium (Eu^3+^) or terbium (Tb^3+^) complexes in water and D_2_O *via* the Horrocks and Supkowski equations. The large size and high molecular weight of the paramagnetic chelate are also determinant parameters to positively affect the rotational correlation time (*τ*_r_) and the residence time of water molecules (*τ*_m_) in the sphere of coordination of the metal.^9–14^ Slower rotation (an increase of *τ*_r_) and faster exchange rate *k*_ex_ (decrease of *τ*_m_, *k*_ex_ = 1/*τ*_m_) improve the relaxivity. Consequently, the diminution of the relaxation time of water protons (T1) is related to a high exchange rate (*k*_ex_). This time *τ*_m_ is dependent on the exchange mechanism, the steric hindrance, and the rigidity of the ligand, as well as the overall charge of the complex. The increase in correlation time and reduction of the rate of rotation of the complex can be obtained by the use of supramolecular molecules such as polymers,^19–21^ dendrimers,^22–24^ micelles,^25–28^ proteins,^29–32^ or nanoparticles.^33–35^ Moreover, an excessive exchange can limit the contact between the nuclear spin of the proton and the electronic spin of the metal and can also lead to a decrease in relaxivity. The residence time of the water molecule in the sphere for an optimal efficiency is between 10 and 100 nanoseconds.

The relaxation of the inner sphere of a contrast agent is also strongly related to the Gd–H distance according to the term (1/*r*_6_). These distances range from 2.7 to 3.3 Å.^36^ Indeed a decrease in the angle of inclination between the bound water molecule and the metal–oxygen bond of 0.1 Å results in a 20% of the increase in relaxivity. However, the achievement of such structural changes is difficult because the exact determination of the Gd–H distance remains complex. Unlike the rotational correlation time that governs relaxivity at strong fields, the electronic relaxation time *τ*_s_, characterizes the relaxivity obtained for weak fields (0.2 Tesla). A slow electronic relaxation induces high relaxivity. However this parameter is very difficult to measure and for the magnetic field used clinically, this value becomes negligible. ^1^H NMRD (Nuclear Magnetic Relaxation Dispersion), ^17^O NMR, and EPR (Electronic Paramagnetic Resonance) are complementary experiments to investigate the properties of magnetic compounds.^37,38^ NMRD profiles are based on the measurement of the longitudinal relaxation rate 1/T1 as a function of the magnetic field strength at various temperature and provide reliable estimates of *τ*_m_ (the water exchange rate), *τ*_r_ (the reorientational correlation time), and *τ*_s_ (the electronic spin relaxation time). The shift of oxygen atom binding to Gd(iii) observed by ^17^O NMR spectroscopy in the function of the temperature and the magnetic field led to the hydration number value, but also to the rotational correlation time and the electronic relaxation rate. The exchange rate between the water molecules with the medium can be also measured and the mechanism elucidated. The electronic relaxation time can be determined by EPR at several magnetic fields. Finally, the contrast agent has to fill few criteria for *in vivo* applications, such as an osmolality close to plasma for a good biological tolerance (0.3 osmol kg^−1^)^39^ and a high stability to avoid the release of Gd(iii) into the organism (pGd). This stability is related to the dissociation constant *k*_d_ of the complex and consequently to the thermodynamic constant *K*_GdL_ corresponding to the affinity of Gd(iii) with the ligand.^9,14^ The MRI tools must be kinetically inert to avoid *in vivo* transmetalation with endogenous Cu(ii) or Zn(ii) anions, for instance. Generally, the half-live time of DOTA(Gd) in the human body is around 90 minutes.

MRI has extensive applications in the diagnosis of neurological, cardiovascular, and oncological diseases. Current contrast agents suffer from non-specificity, rapid renal excretion, and low relaxivity. Consequently, the challenge in the MRI research field is to reach a potent CA that can fill successfully parameters such as high relaxivity value to reach good contrast imaging with strong coordinating ligands to avoid the release of free Gd(iii) into the bloodstream and, high sensitivity to extend the applications to the detection of low concentrated biological targets, typically 10^−9^ to 10^−13^ mol g^−1^ in cell tissue with stable relaxivity in clinical and animal imaging experimental conditions. Indeed, a decrease of relaxivity can occur at high field (4.7 T) and high temperature (37 °C) during *in vivo* experiments. A CA with such features could be injected at a lower concentration to the patient (actually 50–100 mM injection, 0.1 to 0.3 mmol kg^−1^), could limit the repeated injections, could shorter the examination time (less than 30 minutes) improving the comfort of the patient and the acquisition time to reduce the cost of the experiment. And finally, this CA of new generation could reach selectively the tissue, could be internalized into cells *via* carriers with longer residence time to improve the pharmacokinetic and, good renal filtration and low organ accumulation. Consequently, to design the CA four main parameters can be modulated: (i) the ligands obtained with straight forward synthesis, biocompatible, with a rigid spacer to reduce the flexibility; (ii) a high Gd^3+^ loading to improve the contrast and the sensitivity; (iii) a high molecular weight to increase *τ*_r_ and, (iv) a presence of hydrogen bonding to improve the water exchange rate. Research efforts were therefore focused to CA chelates based on bulky macromolecules (polysaccharides, polymers, dendrimers) and/or exploiting self-assembly based on supramolecular interactions. We focused our report on cyclodextrins, a scaffold selected because it can be highly chemically modifiable. Such molecules have an important molecular weight and an adequate water-solubility. They are able to form hydrogen bonding interactions, and already used as host molecule for biomedical applications.

**Fig. 2:**
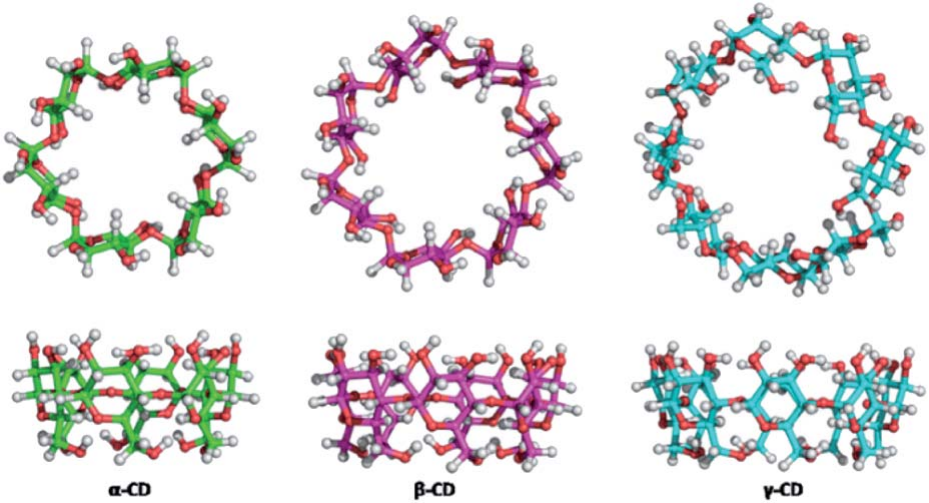
Structures of the different cyclodextrins.

Cyclodextrins (CDs), natural caged molecules, are cyclic truncated oligosaccharides resulting from the degradation of starch by cyclodextrin glucosyltransferase (CGTase).^40–43^ Cyclodextrins contain six to twelve units of glucose. Each unit of d-glucose in chair conformation is bound by its anomeric carbon to the adjacent unit by α-(1–4) glucosidic bonds. α-, β- And γ-cyclodextrins respectively formed of 6, 7 or 8 carbohydrate units are the three families of CDs mainly studied and used (Fig. 2). These macrocycles have a truncated cone shape with two different sides.

**Fig. 3:**
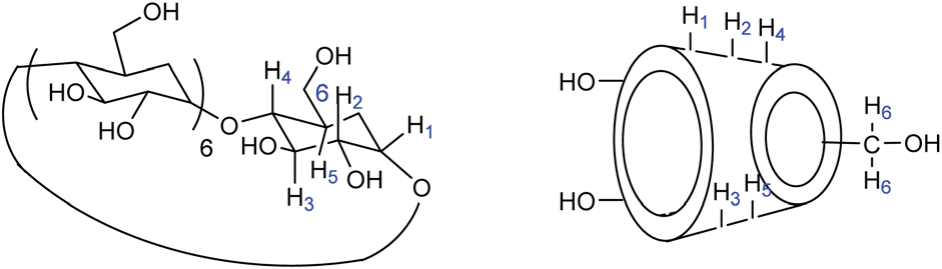
Cyclodextrins nomenclature.

The secondary face is represented by the widest edge, occupied by the secondary hydroxyl groups (in positions 2 and 3) of the glucose units, while the primary face is formed by the narrowest edge, occupied by the primary hydroxyl groups (position 6). The protons H-3 and H-5 are located inside the cavity while the protons H-2, H-4 and H-6 are positioned outside (Fig. 3).

The external surface is more hydrophilic than the internal cavity. Thus, CDs are soluble in water and aprotic polar solvents such as dimethylsulfoxide (DMSO) and dimethylformamide (DMF). The main physicochemical characteristics of the three most studied CDs are represented in the following Table 1.

**Table 1:** Characteristics of cyclodextrins

Types	Number of units glucose	Molar mass (g mol^−1^)	Internal diameter (Å)	External diameter (Å)	Solubility at 25 °C (g per 100 mL of water)
α	6	973	4.7–5.3	14.6	14.50
β	7	1135	6.0–6.5	15.4	1.85
γ	8	1297	7.5–8.3	17.5	23.90

The cavity delimited by the truncated structure is able to include hydrophobic molecules in an aqueous medium. It is one of the most important properties of these macromolecules since it allows the formation of inclusion complexes with a wide variety of organic guests for biological applications.^44–48^ It occurs a dynamic balance between the guest molecule I and the host cyclodextrin (CD). The formation of the complex, as well as its dissociation are governed by thermodynamic laws:

4 [CD] + [I] ⇋ [CD × I]

5 *K*_a_ = [CD × I]/[CD][I]

This process is reversible and can be quantified by an equilibrium constant called association constant *K*_a_ or by a dissociation constant *K*_d_ = 1/*K*_a_ where [CD–I], [CD] and [I] are the concentrations of inclusion complex, host and guest molecules, respectively. One or two molecules can be encapsulated in a non-covalent manner in one or two cyclodextrins, but the most frequent stoichiometry encountered is 1 : 1. This host–guest inclusion complex is governed by weak interactions such as van der Waals, hydrophobic interactions, dipole–dipole. The formation of such inclusion complex can be confirmed and quantified (stoichiometry and stability constant) by various and complementary experiments such as UV-visible and infrared spectroscopies, Nuclear Magnetic Resonance (NMR), calorimetric analysis differential, thermogravimetry, fluorescence, dichroism or isothermal titration calorimetry. This basket-shaped topology with an ‘‘inner–outer’’ amphiphilic character is mainly used to improve the solubility of lipophilic drugs, their delivery and as support for analytical separation.^49–54^

In MRI field, there are two strategies to take advantage of the properties of CDs in order to improve the image contrast. The first one is to form a noncovalent host–guest inclusion complex with a modified contrast agent, the second one is to immobilize covalently the contrast agent on the macromolecule. For this second purpose, several paths exist to functionalize cyclodextrins, however mostly the mono or persubstitutions of the primary alcohol were described for this application. The first step of substitution of the C6-position occurs with many potent electrophiles such as alkyl, phosphoryl, silyl, and sulfonyl halides, sodium triazide, or carboxylic acid chlorides. From these active sites, usual chemistry can be performed to introduce modified MRI contrast agents. The selective chemical modification of the secondary face is based on the difference in acidity between the hydroxyls of the small crown (position 6, p*K*_a_ = 15) and those of the large crown (positions 2 and 3, p*K*_a_ = 12–13). The secondary alcohols in position 3 turn inwards to the cavity while the ones in position 2 points outwards making this position more accessible.

Wong^55^ and Albert^56^ reviewed examples of cyclodextrin-based molecular imaging probes such as hyperpolarized, luminescent, ionizing radiation, ultrasound, photoacoustics, radiolabeled, multimodal and, MRI. This review is focused on the state of the art of Gd(iii) MRI T1 contrast agents based on cyclodextrin scaffolds. In the race of the development of new theranostic probes, more and more sophisticated supramolecular designs are currently reported to target and visualize cancer tumors and other diseases with success proving the great potential of this natural macrocycle.

## 2. Noncovalent host–guest inclusion complexes

To increase the stability constants of the complexes formed, aromatic, cyclohexyl or adamantane groups, known for their strong affinities for the host CD cavities, were used as the anchor of guest Gd(iii) ligands. The effect on MRI signal of the mix of these functionalized contrast agents with one or more cyclodextrins was tested to improve the design and the efficiency of these imaging probes. Thus, Aime *et al.* were the first who functionalized contrast agents such as DOTA-Gd and DTPA-Gd by forming host–guest inclusion complexes between various CDs and polymer of CDs.

### 2.1 Aromatic/aliphatic modified contrast agents

In 1991, Aime *et al.* reported the effect of the formation of an inclusion complex between gadolinium contrast agent and β-cyclodextrin.^57^ For this purpose DTPA and DOTA ligands were modified to introduce lipophilic benzyloxymethyl group (–CH_2_OCH_2_Ph = BOM) able to be included into the cyclodextrin cavity (Table 2). In both cases, there is a single coordinated water molecule (*q* = 1), a similar distance from Gd(iii) around 3.6 Å and a residence time *τ*_m_ of 5 ns. Equilibrium constants were determined for the first time by NMR titrations and estimated at 2.8 × 10^2^ M^−1^ and 2.2 × 10^2^ M^−1^ for complexes DOTA(BOM-H-BOM) and DTPA(BOM), respectively. The interactions between BOM units in excess of β-CD were highlighted by comparison with DOTA-Gd alone. Unmodified DOTA, in the presence of CD, even in excess, does not influence the relaxation rate of the contrast agent. Furthermore, while a variation of *r*_1_ is observed for complexes based on DTPA ligand in the presence of β-CD, the relaxation remains constant in presence of β-CD with a smaller cavity that prevents the formation of an inclusion complex. The other cases underlined that the host–guest interactions generated an elongation of the molecular reorientational *t*_r_ which results in an increase of solvent proton relaxation rates. As 10 fold excess of hosts was used, titration results showed for contrast agent based on DTPA modified with two aromatic moieties were included into two cyclodextrins cavities.

**Table 2:** Structure of the different modified DOTA and DTPA ligands

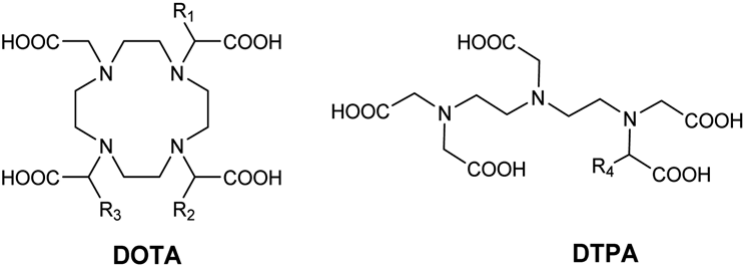			
R_1_	R_2_	R_3_	R_4_
H	H	H	H
BOM	H	H	BOM
BOM	H	BOM	—
BOM	BOM	H	—
BOM	BOM	BOM	—

**Fig. 4:**
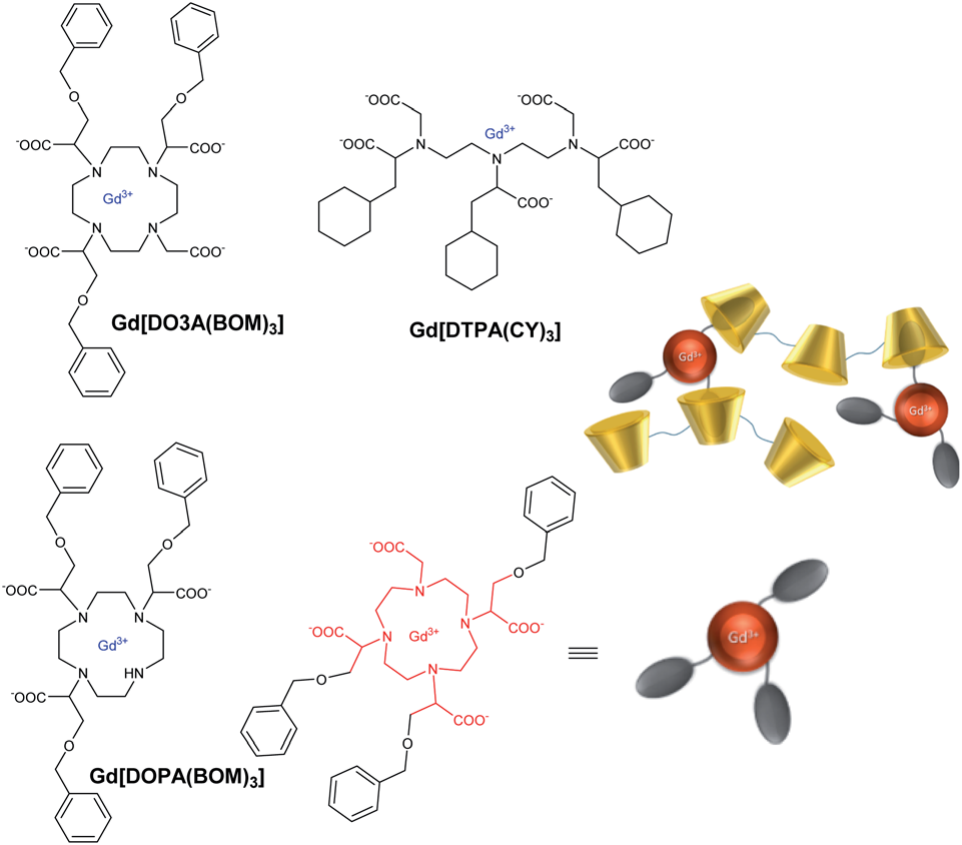
Structures of contrast agents and inclusion complexes in polymer of β-CDs.^59^

A non-covalent adduct was studied using a paramagnetic complex based on heptadentate ligand (*q* = 2) containing a *para*-bromobenzyl linker (Fig. 4).^58^ It led to a relaxivity increase of a factor of 2.1 and 4.1 with β-CD and polymer of CDs obtained by the crosslinking with 1-chloro-2,3-epoxypropane (MW 6 kDa), respectively. The excess of CD cavities favored the intermolecular interactions and the larger size of the polymer caused elongation of the molecular reorientational time *τ*_r_ resulting in a relaxivity enhancement (32 mM^−1^ s^−1^, 20 MHz, 25 °C). A similar study in a blood sample showed that 5% of the paramagnetic complex was bound to HSA with two interaction sites in the subdomains IIA and IIIA of the protein and underlined a potential application in angiographic imaging. The same team synthesized other contrast agents based on tricyclohexyl-DTPA (DTPA(CY)_3_) and tribenzyloxymethyl-DOTA (DO3A(BOM)_3_) and four carboxylate functions (Fig. 4).^59^ The studies have been performed in absence of β-CD, in presence of β-CD, and polymer of β-CDs with a degree of polymerization of 12 (Table 3). The absence of the fourth carboxylic acid function in DO3A(BOM) improved the relaxivity by 20% due to the presence of two water molecules in the inner sphere. An enhancement of a factor of 3 and 6 was observed in presence of β-CDs and poly-β-CDs, respectively. Indeed in presence of polymer, a relaxivity value of 61 mM^−1^ s^−1^ (25 °C, 20 MHz) was reported. The authors suggested that the reinforcement of the hydrogen-bonding in the polymeric network appeared to be responsible for this enhancement thanks to the contribution of water molecules in the second coordination sphere of gadolinium atom. Indeed the crown of hydroxyl groups of the β-CD yields stronger interactions with the water molecules around the complex lengthening their lifetime in the proximity of the paramagnetic center.

**Table 3:** Relaxivities of the free Gd^III^ complexes and in presence of inclusion complexes with β-CD and poly-β-CD

	*r*_1_ [mM^−1^ s^−1^]	*r*_1_ [mM^−1^ s^−1^]	
		β-CD	Poly-β-CD
[Gd(DTPA(CY)_3_)]	9.1	25.0	52.7
[Gd(DOTA(BOM)_3_)]	7.5	25.0	49.0
[Gd(DO3A(BOM)_3_)]	10.0	30.4	61.0

**Fig. 5:**
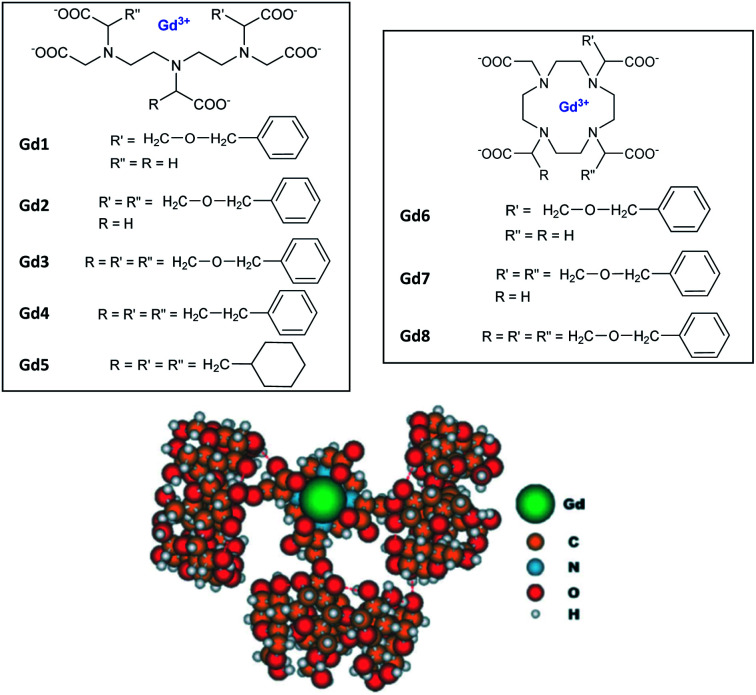
Structures of Gd(iii) complexes derived from DOTA and DTPA ligands and modelisation of inclusion complexes with 3 cyclodextrins. This figure has been adapted/reproduced from ref. 60 with permission from John Wiley & Sons, Inc., copyright 2003.

Aime's group in 2003 further studied other functionalizations with ethylbenzyl and cyclohexyl groups capable of multiple interactions with β-CD cavities (Fig. 5).^60^

Relaxometric titrations were performed to determine the dissociation constants of these octacoordinate Gd(iii) chelates in presence of one (*K*_d1_), two (*K*_d2_) or, three β-cyclodextrins equivalents (*K*_d3_) (Table 4). The relaxation values for the inclusion complexes without (*r*_1_), with one (*r*_1-1_), two (*r*_1-2_) or, three β-cyclodextrins (*r*_1-3_) are summarized in Table 4 below (25 °C, 20 MHz).

**Table 4:** Dissociation constants and relaxivities of the complexes between Gd3–5 and Gd8 contrast agents (Fig. 5) and 3 β-CDs

	*K*_d1_ (10^−5^ M)	*K*_d2_ (10^−3^ M)	*K*_d3_ (10^−2^ M)	*r*_1-1_ (mM^−1^ s^−1^)	*r*_1-2_ (mM^−1^ s^−1^)	*r*_1-3_ (mM^−1^ s^−1^)
Gd3	145	8.33	5.00	11.44	18.48	28.20
Gd4	38.5	2.22	4.00	10.66	17.22	26.24
Gd5	9.1	0.48	1.43	11.37	18.65	28.22
Gd8	73.0	4.54	2.00	12.75	21.75	27.71

**Fig. 6:**
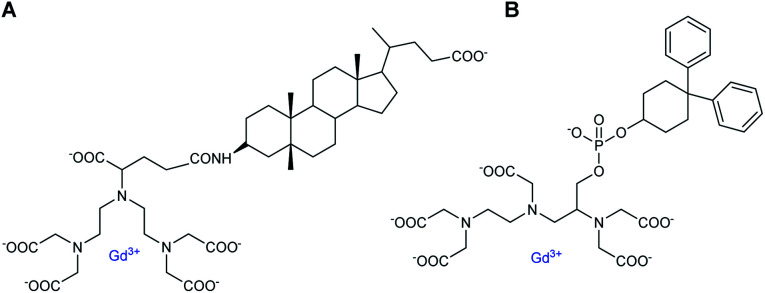
Contrast agents based on DTPA ligand substituted with lithocholic acid (A) and bisphenylcyclohexylphosphate (B) groups.

The increase in number of hosts decreased the strength of the intermolecular interactions from 10^−4^ to 10^−2^ M and caused a raising of the relaxivity around 10 to 28 mM^−1^ s^−1^ with, for instance for Gd8, 12.75/Gd, 10.87/Gd, and 9.23 mM^−1^ s^−1^ per Gd, with one to three cyclodextrins stoichiometries, respectively. As comparison, a relaxivity of 7.5 mM^−1^ s^−1^ (25 °C, 20 MHz) was observed with this ligand without CD.^59^ A linear correlation was observed between the relaxivity values and the molecule size of the studied complexes with no large difference between DTPA and DOTA structures. However, the relaxivity is not function of the nature of the lipophilic ligand and its stability constant. Thus, larger size and mass of the supramolecular adducts involving a trifunctionalized contrast agent and three CDs moieties generated higher relaxivity due to the increase of the overall molecular reorientational time. They reported that a complex with a molecular weight around 4 kDa yielded relaxivity of the order of 25 mM^−1^ s^−1^, five-time higher than the clinical used DOTA. Immobilization of chitosan on the primary face of β- and γ-CDs using a functionalized linker bound by amide and ester functions (20 CDs/chitosan units) was performed.^61^ This cheap water-soluble biopolymer is known for its great biocompatibility and biodegradability. Two guests were tested based on DTPA ligand firstly substituted with a lithocholic acid and secondly with a bisphenylcyclohexylphosphate groups, both known to be recognized by the cavity of β- and γ-CD, respectively (Fig. 6). By comparison with native β- or γ-CD, PRE (Proton Relaxation Enhancement) analysis showed that chitosan adducts had a stronger binding affinity toward the contrast agents (one magnitude order) in presence of biopolymer and led to an enhancement of relaxivity of 104% and 140% with lithocholic acid and phosphate groups, respectively (Table 5). This result confirms the positive impact of these supramolecular vectors on imaging. However, the authors underlined competitive interactions with phosphate ions and blood albumin limiting the potential for *in vivo* applications.

**Table 5:** Binding parameters and relaxivities between contrast agents A and B (Fig. 6) with γ/β-CDs and polymers of γ/β-CDs obtained from the fitting of PRE titrations (pH 7, 298 K, 0.47 T)

Complexes	Lithocholic acid/γ-CD	Bisphenylcyclohexylphosphate/β-CD
*r*_1_, mM^−1^ s^−1^	8.5	7.7
*K*_a_, with CD, 10^4^ M^−1^	9.4	8.0
*r*_1_ with CD, mM^−1^ s^−1^	10.6	11.5
*K*_a_, with polymer, 10^4^ M^−1^	20.1	30.1
*r*_1_ with polymer, mM^−1^ s^−1^	21.6	27.5

**Fig. 7:**
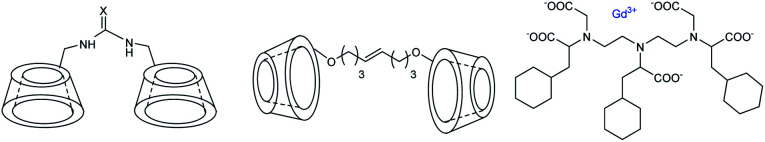
Structures of cyclodextrin dimers and guest molecules.

As the presence of an excess of cyclodextrins improved the relaxivity, Aime *et al.* have tested dimers of CDs covalently linked by urea, thiourea functions, or an aliphatic unsaturated chain (Fig. 7).^62^ Stable host–guest inclusion complexes were formed with functionalized DTPA bearing three cyclohexyl groups.

**Fig. 8:**
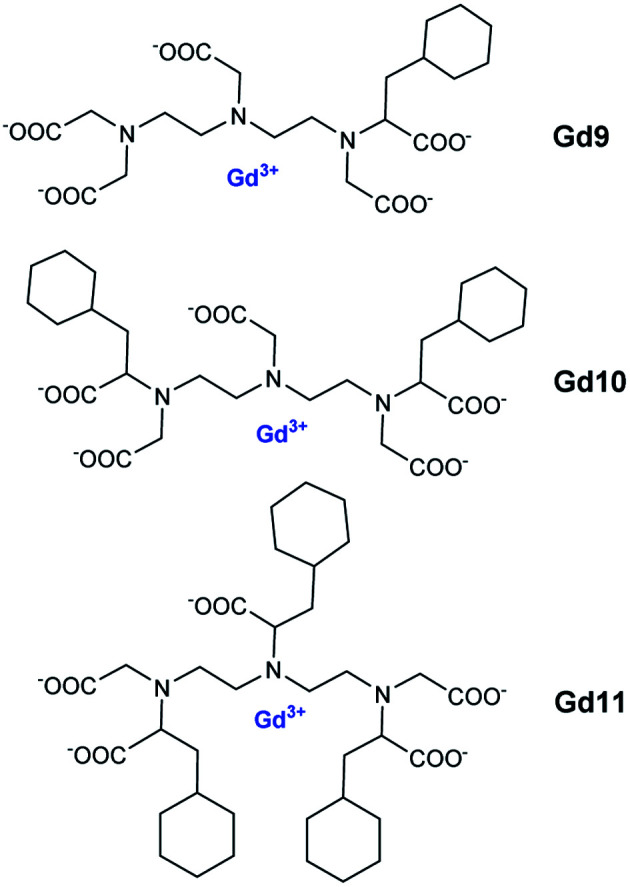
Structure of contrast agents Gd9–11.

**Fig. 9:**
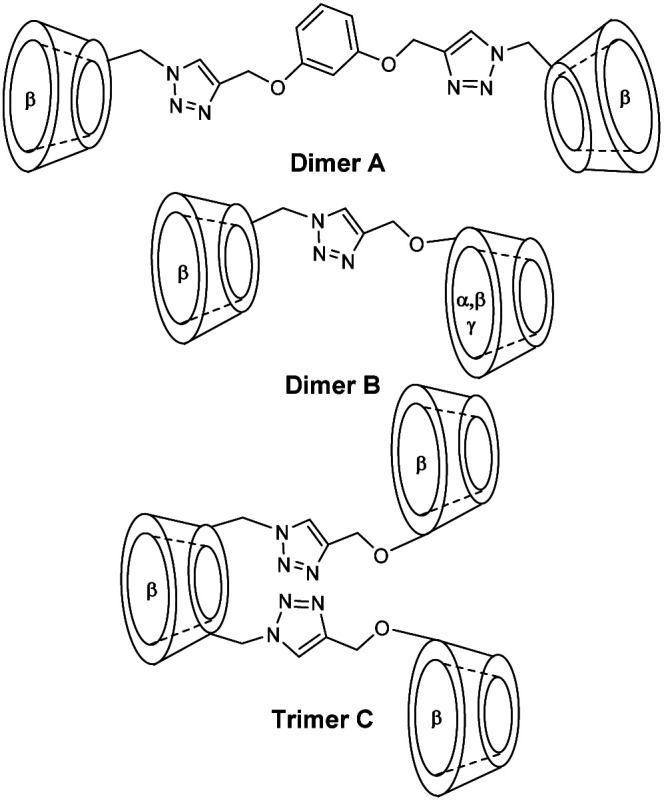
Structure of dimers or trimer of cyclodextrins.

Thus, cooperative supramolecular interactions occurred and the relaxometric studies were enhanced by a factor of 3 and 4 with 9.1 mM^−1^ s^−1^ for β-CD to 27.7 mM^−1^ s^−1^ for 6,6′-ureido and 35 mM^−1^ s^−1^, thioureido-bis-CDs (25 °C, 20 MHz), respectively. Stability constants were also modified between native β-CD (*K*_a_ = 8 × 10^2^ M^−1^) and urea (*K*_a_ = 3.1 × 10^3^ M^−1^) and thioureido-bis-CDs (*K*_a_ = 3.0 × 10^3^ M^−1^). The more flexible dimer with unsaturated chain showed one order of magnitude higher stability constant with 4.3 × 10^4^ M^−1^ and a high relaxivity value of 27.3 mM^−1^ s^−1^ due to a better fitting of cyclohexyl groups into the two CD-cavities. These structures were described as promising candidates as diagnostic markers. Bridged α-, β- and γ-cyclodextrins dimers and trimers using click chemistry at various positions (2 and 6) were prepared (Fig. 8 and 9).^63^ The easy Huisgen 1,3-dipolar cycloaddition in presence of Cu(i) salt allowed the introduction of many linkers with different lengths and functionalizations. Symmetric and unsymmetric triazoles bridged multimers were obtained with good yields with unlike (head to tail) and like faces (head to head). DTPA ligands with one to three cyclohexyl lipophilic moieties were tested. The authors assumed that the ditopic guest molecules would be bound to bis-CDs more strongly than monotopics ones. Affinity constants and relaxivity values were reported and compared (Table 6). The proximity of the cavities allowed the better fitting of the lipophilic moieties of the contrast agents leading to a better supramolecular assembly. Unlike orientation cages with bis-CDs joined head to tail seemed to offer the best combination. The intermolecular interactions with Gd(iii) chelates with dimer or trimer of cyclodextrins were relatively stable, association constants ranging from 4.6 × 10^3^ M^−1^ to 5.3 × 10^4^ M^−1^ being reported. The adducts showed higher stabilities of 2 to 3 orders of magnitude than those formed with native CD. As expected, better imaging properties were observed with trimer adducts (*r*_1_ = 35 mM^−1^ s^−1^) due to tritopic complexes that act as bridges linking two or three dimer molecules leading to a longer *τ*_r_ value resulting from larger dimension of the supramolecular complex. The simple inner–outer sphere model was used but the contribution of the second-coordination sphere was also demonstrated. The impact of the presence of multicavities of cyclodextrins pinpointed the importance of noncovalent interactions in the design of a new MRI contrast agent endowed with markedly better contrasted images.

**Table 6:** Binding parameters and relaxivities determined by the analysis of NMRD profiles (Fig. 8)^a^

	Gd9	Gd10	Gd11
Dimer A	*K*_a_ = 5.9 × 10^3^	*K*_a_ = 5.9 × 10^3^	—
	*r*_1_ = 11.5	*r*_1_ = 25.8	—
Trimer C	*K*_a_ = 1.4 × 10^4^	*K*_a_ = 5.3 × 10^4^	*K*_a_ = 6.1 × 10^3^
	*r*_1_ = 12.8	*r*_1_ = 27	*r*_1_ = 35.6
	*q* = 0	*q* = 5 ± 1	*q* = 9 ± 1.5

a *K*_a_ = association constant (M^−1^), *r*_1_ = adduct relaxivity (mM^−1^ s^−1^, 25 °C, 20 MHz), *q* = number of second-sphere water molecules.

### 2.2 Biomedical applications

#### 2.2.1 Nanoparticles

**Fig. 10:**
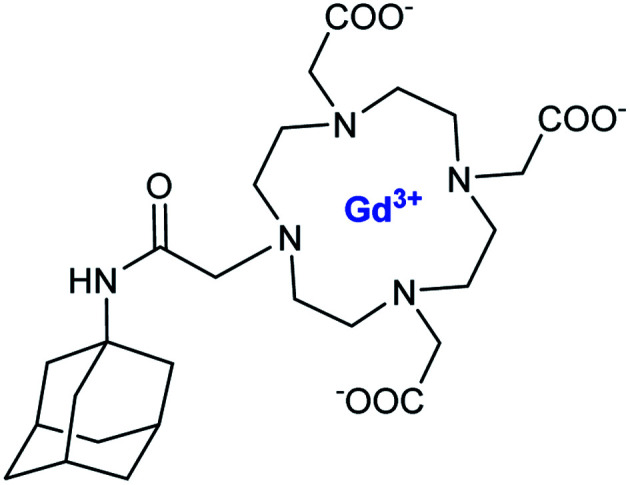
Structure of adamantane-DOTA.

From 2006, this lock-and-key recognition concept was extended with a new noncovalent adduct using adamantane group, known for its high affinity with cyclodextrin cavity. An adamantane-DOTA ligand was synthesized using acetamide function (Fig. 10). This chelate is stable in the range of pH 4–12 and has a relaxivity of 5.2 mM^−1^ s^−1^. This new functionalized contrast agent was successfully tested in presence of β-CD and of high molecular weight polymer of β-CDs (average MW of 6–130 kDa).^64^ Indeed, high stability constant were measured by proton relaxation enhancement (PRE) method, and values of 4.9 × 10^3^ M^−1^ and 6.3 × 10^3^ M^−1^ for β-CD and its polymer, respectively were obtained (Table 7). The relaxometric characterization of the adducts led to a relaxation enhancement of 54% in presence of the polymer of CDs (14.8 mM^−1^ s^−1^ to 20 MHz and 37 °C) by comparison with β-CD alone (9.6 mM^−1^ s^−1^) due to higher loading of Gd(iii) and molecular weight. For linear polymer above MW 10 kDa, no correlation between rotational time and molecular weight occurred.

**Table 7:** Binding parameters and relaxivities of supramolecular adducts with β-CD and polymer of β-CD

Host	*K*_a_ (M^−1^)	*r*_1_ (mM^−1^ s^−1^)
β-CD	4.9	9.6
Pβ-CD	6.3	14.8

**Fig. 11:**
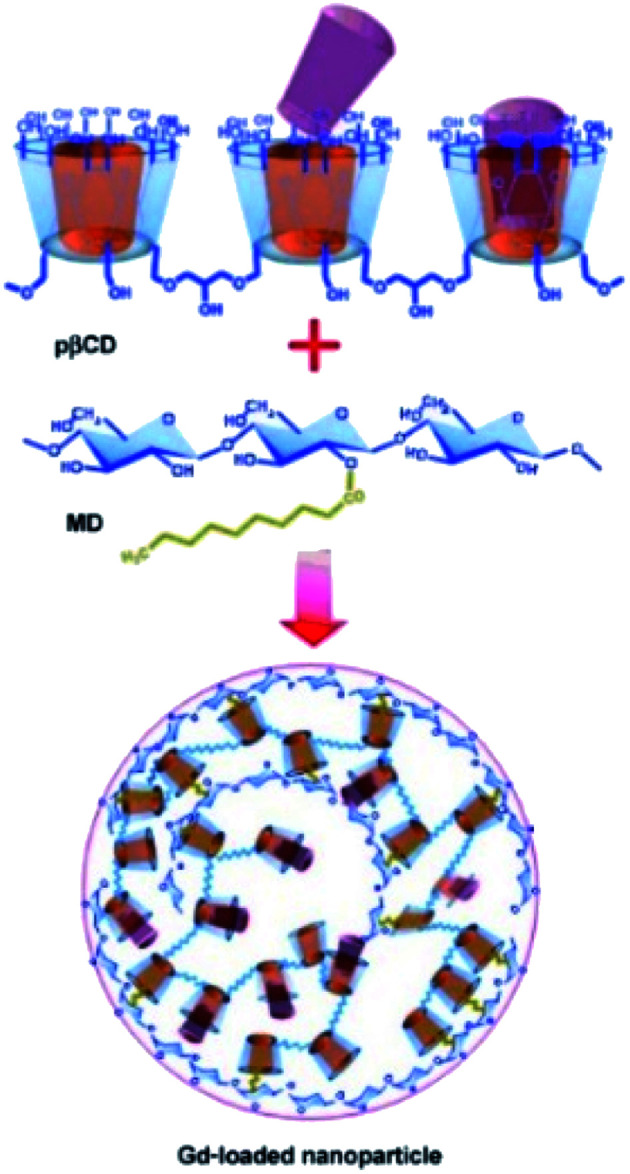
Formation of Gd^III^-loaded nanoparticles through a supramolecular three-component assembly. This figure has been adapted/reproduced from ref. 64 with permission from John Wiley & Sons, Inc., copyright 2008.

From these results, new engineered supramolecular nanoassemblies were developed and 200 nm diameter nanoparticles using dextran grafted with alkyl chain allowing to entrap 1.87 × 10^5^ molecules of Gd chelates were isolated (Fig. 11).^64^ This nanosystem was prepared in a one-step procedure without solvent of surfactants leading to high Gd(iii) loading and high water molecules density (70 wt% of the nanogel is water) surrounding the lanthanide. A great relaxivity (33.5 mM^−1^ s^−1^ at 20 MHz and 25 °C) was observed. An enhancement to 48.4 mM^−1^ s^−1^ was reached at physiological temperature (37 °C) due to the increase of the water exchange rate while Gd(iii) amount and size of the nanoparticle being constant between these two temperature conditions. This strategy seemed to be promising for the *in vivo* targeted delivery of Gd(iii) complexes. Indeed, this macromolecular Gd-based system was stable over 36 hours and compatible with intravenous administration. Furthermore, it was expected to be less toxic due to the absence of covalent links with Gd(iii) chelate making easier its bioelimination.

**Fig. 12:**
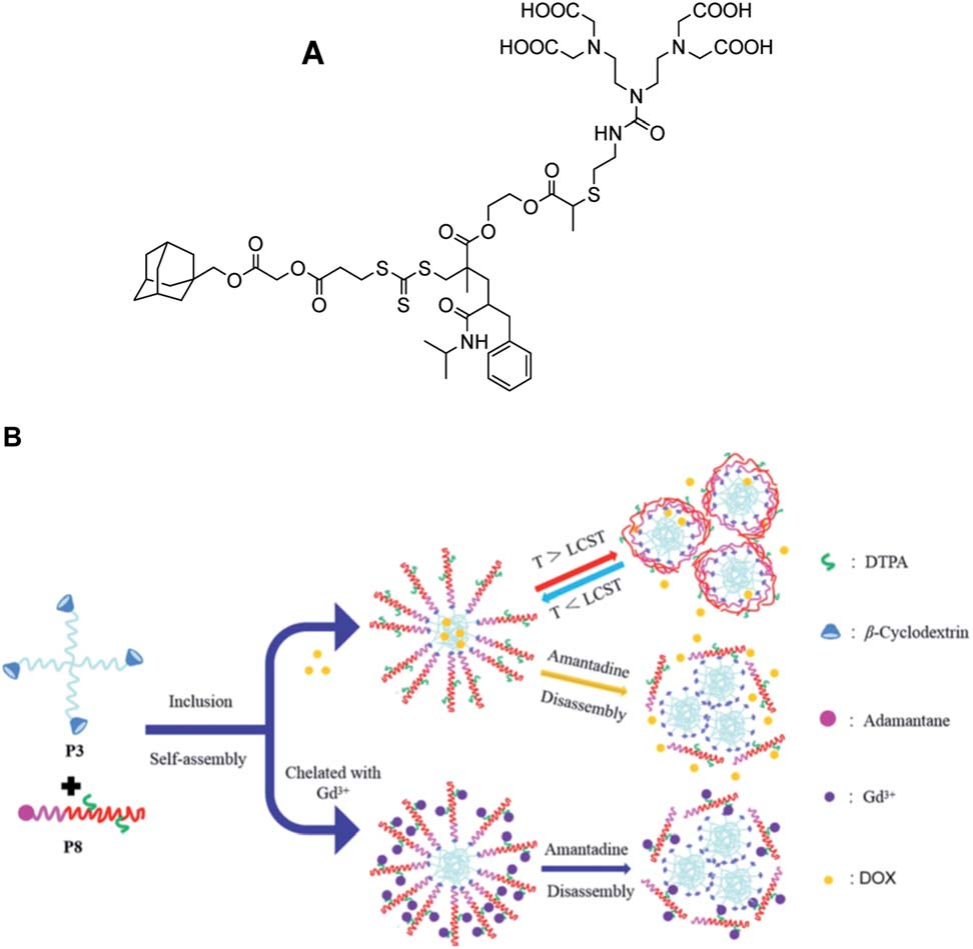
(A) Figure of adamantine-DTPA-Gd ligand. (B) Schematic illustration for self-assembly and disassembly of supramolecular polymer and application in MRI and drug-carrying. This figure has been adapted/reproduced from ref. 65 with permission from Elsevier, copyright 2020.

In 2020, Zhou and his group designed self-assembly of stimulus-responsive star-shaped copolymer using two polymers based on β-CD (host) and adamantine-DTPA-Gd ligands (guest) (Fig. 12).^65^ β-CD was functionalized using click chemistry on polycaprolactone. A derivative of poly-l-lactic-*block*-poly(methacrylate) was used to immobilized adamantine by ROP and RAFT reactions. The amphiphilicity properties of the host–guest inclusion complex between the adamantane group and the cavity of cyclodextrin led to monodisperse spherical micelles (90 kDa, 296 nm) with a CMC (Critical Micelle Concentration) of 36 mg L^−1^. A strong proton correlation in 2D ^1^H NOESY (Nuclear Overhauser Effect) spectra proved the formation of the inclusion complex. Surprisingly, similar relaxivity to Magnevist® was observed. *In vitro*, it was proved that a competitive guest inclusion complex using an external 2-adamantylamine molecule disassembled the nanostructure reducing its molecular size. The stability of the micelle with temperature was studied using doxorobucin as a drug marker. A faster release was observed in the following order with the addition of external guest, at 25 °C and 37 °C, respectively with the apparition of aggregates of the dis-assembly polymers. This temperature-responsive strategy is suggested to control drug release.

#### 2.2.2 Bimodal probes

The research has been focused on the synergic effect of two imaging probes as MRI, fluorescent, near-infrared or, ^19^F-MR on the same structure to cumulate a high resolution and a high sensitivity. Such bimodal association can improve the performance and generates more accurate information about the disease.

**Fig. 13:**
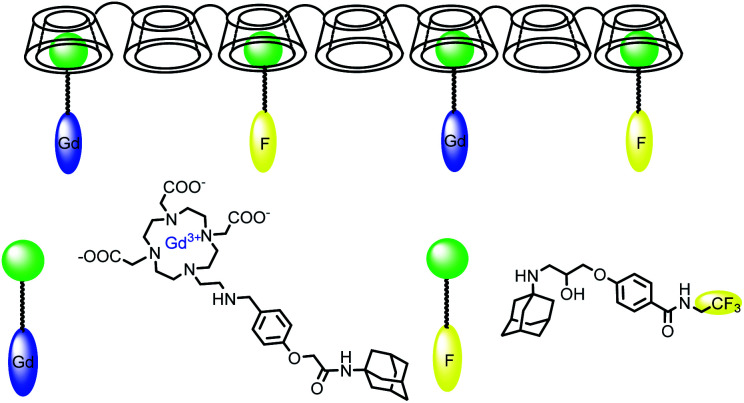
Structure of the supramolecular adduct between poly-β-CD, the Gd(iii)-complex and the ^19^F molecule.

**Fig. 14:**
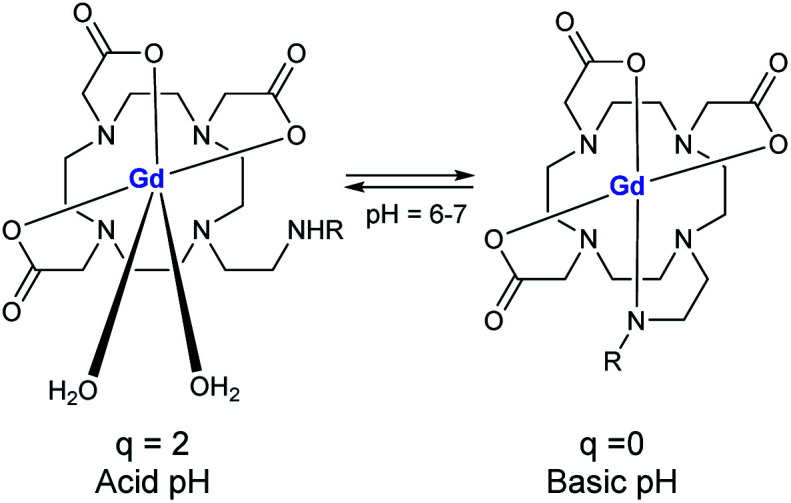
Mechanism of action of the pH-responsive.

In 2009, Aime developed a supramolecular poly-β-cyclodextrin-^19^F-Gd adduct to validate an *in vitro* MRI pH mapping method (Fig. 13).^66^ Indeed, pH changes are often associated with the emergence of various pathologies such as tumors, strokes, and infections. Fluorine and Gd(iii) were both beared by an adamantane anchor equiped with an aromatic linker to assure inclusion complexes with poly-β-CDs (pCD) (8–10 β-CD units). Binding affinities were estimated by PRE method at 1.3 × 10^3^ M^−1^ for Gd/pCD and ^1^H NMR experiment at 1.4 × 10^4^ M^−1^ for ^19^F/pCD, respectively. The authors assumed that such design kept far enough ^19^F nuclei from the Gd(iii) center and consequently avoid the possible drawbacks in the signal acquisition. The pH responsiveness was provided thanks to a sulfonamide arm introduced on a DOTA chelate. From pH = 6.7 the deprotonation of the sulfonamide led to an intramolecular complexation of the paramagnetic lanthanide replacing its inner-sphere water molecules and reducing the relaxation rate (Fig. 14). In complete inclusion complex conditions (pCD : ^19^F-reporter : Gd-complex = 20 : 5 : 1), *in vitro* acquisition of ^1^H and ^19^F-MR images of a phantom were performed and intensity of image was improved at basic pH.

**Fig. 15:**
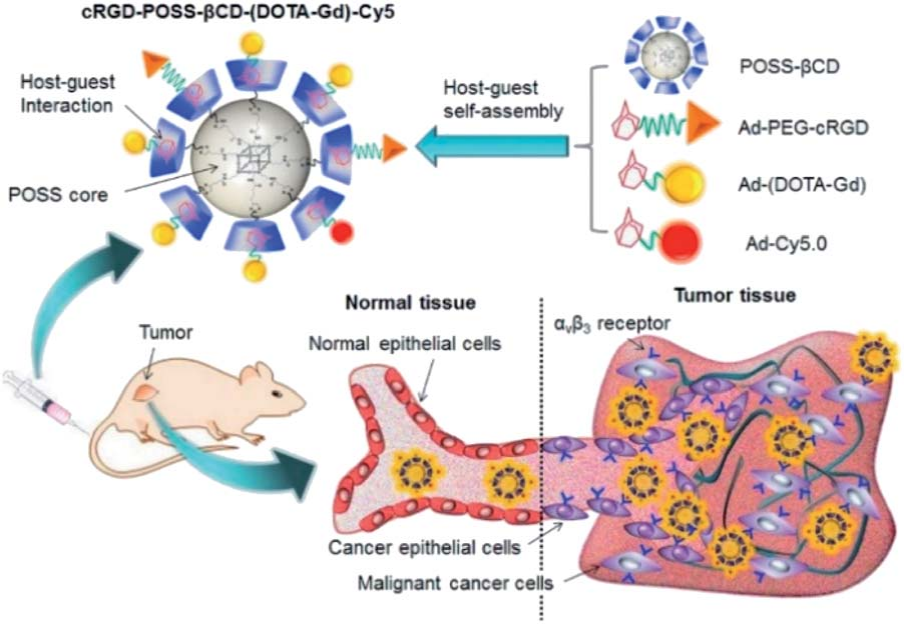
Schematic representation and mechanism of action of the bimodal probe for breast cancer detection. This figure has been adapted/reproduced from ref. 67 with permission from Elsevier, copyright 2016.

In 2017, Lu *et al.* reported an innovative cyclodextrin-based nanoglobular contrast agent from host–guest self-assembly for targeting *in vivo* MR cancer molecular imaging (Fig. 15).^67^ 5 to 8 β-CD units were attached using click chemistry to biocompatible POSS (polyhedral oligomeric silsesquioxane) platform forming a host nanoglobule. A molecular weight around 10 kDa with 7.2 β-CD per POSS molecule was obtained. Adamantane anchor was selected to vectorize guests molecules such as an MRI contrast agent (DOTA ligand), a fluorescent probe (Cy5), and an active cyclic RGDfK peptide (cRGD) used at the biological target. The relaxivities of Ad-(DOTA-Gd), β-CD-(DOTA-Gd), and POSS-β-CD-(DOTA-Gd) at 1.5 T and 37 °C were measured at 3.17, 6.36, 9.50 mM^−1^ s^−1^, respectively. The presence of other guests (Cy5, cRGD) did not affect the signal, the value remaining constant in presence of cRGD-POSS-β-CD-(DOTA-Gd)-Cy5. *In vivo* imaging were carried out and a stronger contrast image was observed (1.5 fold in 10 minutes post-injection) in the tumor with the smart probe in comparison with the control agent with a better concentration in the periphery area than in the tumor interior due to a better angiogenic vasculatures environment. The signal regularly decreased over time probably to a slow host–guest dissociation process and predicting a future rapid excretion by renal filtration and minimizing the possible toxic side effects. Consequently, this biomarker expressed on cancer cell surface generated sufficient signal enhancement for detection. Such specific interaction was confirmed by histological studies. This safe and effective targeted nanosized MRI contrast agent is a promising concept for cancer molecular imaging.

**Fig. 16:**
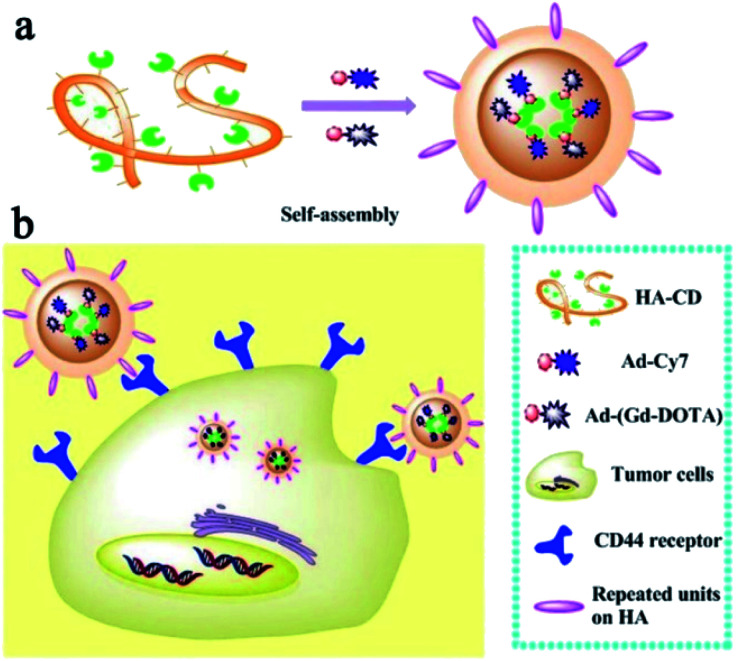
(a) Schematic illustration of bimodal MRI and NIR HA–CD–GC NPs self-assembled *via* a inclusion complex of β-cyclodextrin with modified Ad–Gd–DOTA and Ad–Cy7. (b) Schematic illustration of HA–CD–GC NPs as tumor targeted imaging probes, internalize into the cytoplasm *via* CD44 receptors. This figure has been adapted/reproduced from ref. 68 with permission from Elsevier, copyright 2017.

Sun *et al.* in 2017 reported a multimodal imaging probe based on self-assembled supramolecular nanoparticle combining magnetic resonance imaging (MRI) and near-infrared imaging (NIR) for tumor diagnosis (Fig. 16).^68^ The strategy was based on host–guest interactions between hyaluronic acid and β-cyclodextrin (HA–CD) and two guests (Gd–DOTA (G) and cyanine dye Cy7 (C)) both substituted with adamantane (Ad) group. Hyaluronic acid is biocompatibility, biodegradability, and non-immunogenicity polysaccharide present in the extracellular matrix and joints. It interacts with hyaluronan receptors (CD44) overexpressed in some tumor cells. Irregularly granular were obtained with a size around 30–60 nm. The nanoparticle HA–CD–GC exhibited a relaxivity of 11.4 mM^−1^ s^−1^ (0.5 T) and excellent fluorescence properties. The relaxometric properties were mainly due to the loading of the Gd(iii) chelate and the high local density of water molecules bearing hydroxyl groups on the HA chain. The nanoparticles were biocompatible and non-toxic. Their internalization into tumor cells *via* HA-receptor CD44-mediated endocytosis proved the efficiency of this supramolecular association for imaging of biomarker.

#### 2.2.3 Stimuli-sensitive MRI probes

The first stimuli-sensitive MRI probe was described by Aime and his team in 2007. A tetraazacyclododecanetriacetic acid DO3AS(Gd) complex was functionalized by a 2-pyridyl-dithio group *via* a diacetamido flexible linker.^69^ This contrast agent bearing two water molecules in its inner sphere showed a high relaxivity of 8.1 mM^−1^ s^−1^. The aromatic group can form an inclusion complex with the β-cyclodextrin and polymer of β-CD (30 kDa) cavities leading to an elongation of the correlation time for molecular reorientation motions with relaxivities of 9.2 mM^−1^ s^−1^ and 10.8 mM^−1^ s^−1^, respectively (at 20 MHz, 25 °C and pH 7.4). In presence of glutathione (GSH) the initial disulfide bond was reduced, a new thiol–glutathione bond was formed and the lipophilic aromatic group was released inhibiting any inclusion complex of the CA into the β-CD cavities. A drop of the MRI signal to 47–60% in the absence and presence of β-CD was observed. To explain this reduction, the authors suggested an intramolecular coordination of the inner-sphere of the Gd(iii) by the carboxylate function of the glutathione reducing the hydration center. This hypothesis was confirmed with the ^1^H-NMR dispersion profiles and the ^17^O-NMR transverse relaxation time *versus* temperature profile.

**Fig. 17:**
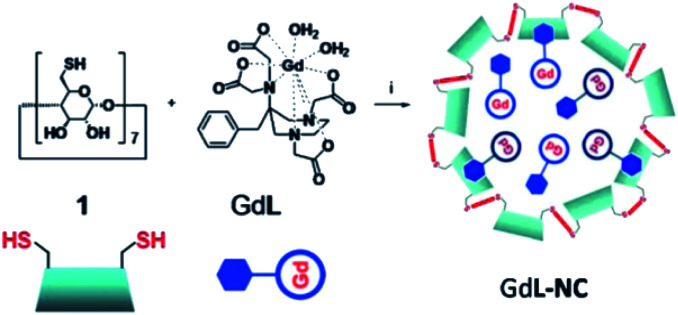
Synthesis of the nanoparticle GdL-NC. Reproduced from ref. 70 with permission from the Royal Society of Chemistry.

Botta *et al.* in 2011 reported perthiolated β-cyclodextrin-based nanocapsules (235 nm) incorporating high loading of bishydrated Gd(iii)-complexes bearing AAZTA arms with benzyl hydrophobic pendant to form host–guest inclusion complexes (Fig. 17).^70^ The binding interaction which represents the driving force for an efficient encapsulation of the Gd(iii) complex into the nanoparticles was estimated at 10^3^ M^−1^ and the concentration ratio at 6 β-CD per Gd ligand.

**Fig. 18:**
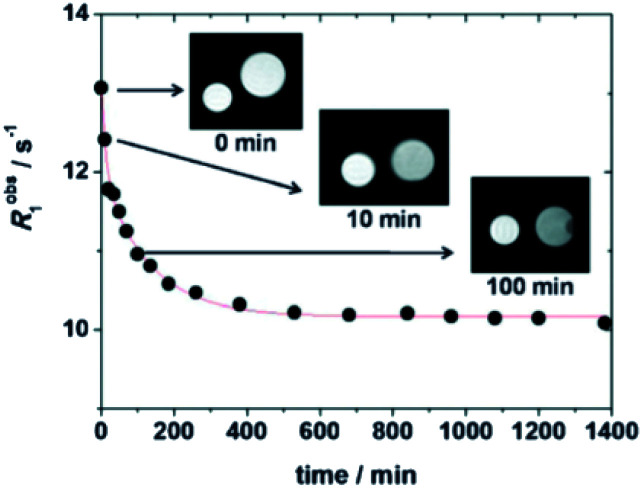
MR images of degradation of GdL-NC at 37 °C and 1.0 T. This figure has been adapted/reproduced from ref. 70 with permission from Royal Society of Chemistry, copyright 2011.

The relaxivity for the nanocapsule (19.3 mM^−1^ s^−1^ per Gd, 20 MHz) was found much higher than that of the free contrast agent (7.3 mM^−1^ s^−1^) in the presence of a six-fold excess of β-CDs (11.9 mM^−1^ s^−1^) because of the high molecular weight of the nanosystem. Indeed the restricted molecular mobility of paramagnetic probe encapsulated inside the particle, and the high permeability of water through the polymeric shell improved the access of the water molecules to the paramagnetic metal centers. The solution of the capsules was stable for several days at room temperature and neutral pH. This perthiolated nanosized system being sensitive to redox status, the cleavage of the eight disulfide bridges occurred in a reducing environment. As the process was not spontaneous, a plateau was reached in about 10 hours. The release of the free paramagnetic complexes and CD monomers caused an increase of the rotational mobility (*τ*_r_) and a specific relaxivity reduction of 20% of the signal (15.5 mM^−1^ s^−1^). The visualization of the kinetic reaction has been possible thanks to a phantom-imaging experiment; *i.e.* MRI signal intensity decreasing with time (Fig. 18). This activable redox responsive nanosystem opened the way to the development of new smart MRI probes.

**Fig. 19:**
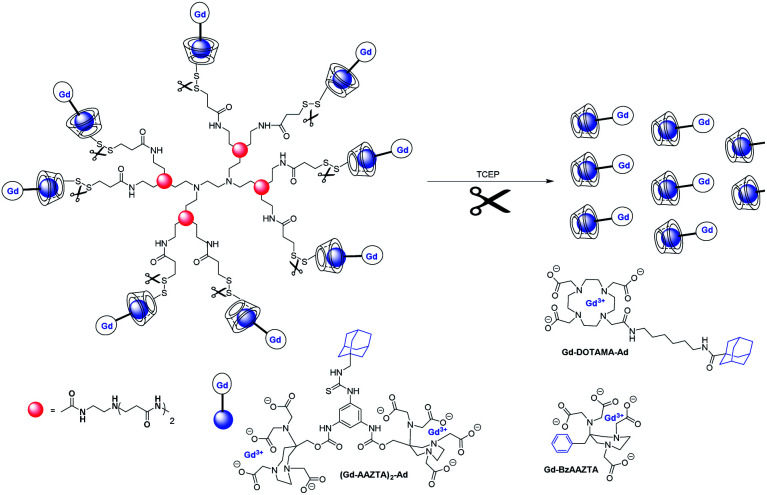
Cleavage by reduction of the disulfide bonds that connect the CD units to the PAMAM scaffold with tris(2-carboxyethyl)phosphine (TCEP).

Botta, in 2014, published a ditopic Gd(iii) complex for the preparation of supramolecular assemblies with β-cyclodextrin, poly-β-CD, and human serum albumin (HSA) (Fig. 19).^71^ Two Gd-AAZTA-like units were linked to a phenyl-ring core functionalized with an arm bearing an adamantyl moiety. This compact and rigid bimetallic Gd(iii) chelate with two water molecules in its inner coordination sphere led to relaxivity of 16.7 mM^−1^ s^−1^ (298 K, 40 MHz) and remains constant over a large pH range (4–11). An expected relaxation enhancement was observed of 28%, 103% and 148% in presence of β-CD (21.4 mM^−1^ s^−1^, *K*_a_ = 1.05 × 10^4^ M^−1^), poly-β-CD (15 kDa, 13 β-CD units, 33.9 mM^−1^ s^−1^, *K*_a_ = 1.27 × 10^4^ M^−1^), and HSA (41.4 mM^−1^ s^−1^, *K*_a_ = 1.20 × 10^4^ M^−1^), respectively which reflects the slowing down of molecular tumbling. *In vivo*, MRI application in tumor bearing mice of ditopic contrast agent alone showed an enhancement of 160% of the signal after one hour from the injection, and a weak hepatic accumulation. The thiol/disulfide redox couple present in tumor tissues was also exploited using a new macromolecular host composed of eight β-cyclodextrins units attached to poly(amidoamine) (PAMAM) dendrimer through a disulfide bond cleavable under reducing conditions.^72^ A ^1^H NMR relaxometric investigation was performed on non-covalent inclusion complexes with three Gd(iii) chelates of mono and ditopics AAZTA (*q* = 2) or DOTA (*q* = 1) functionalized with phenyl or adamantane moieties. Monotopic Gd-AAZTA with pendant benzyl group with low *K*_a_ constant values (3 × 10^2^ M^−1^) had a high value of relaxivity (*r*_1_ = 9.3 mM^−1^ s^−1^) thanks to the presence of two water molecules in the inner-sphere. Indeed, only a relaxivity value of 6.3 mM^−1^ s^−1^ was obtained with octacoordinated DOTA-adamantane chelate. Interactions between Gd(iii) chelates and CD dendrimer were studied with an 8 : 1 ratio. Experiment with benzyl monotopic Gd-AAZTA led to a value of 17 mM^−1^ s^−1^, which corresponds to 82% enhancement whereas very high relaxivity of 456 mM^−1^ s^−1^, corresponding to 28.5 mM^−1^ s^−1^ per Gd ion was detected for adamantane-ditopic ligand with a much stronger affinity (2.3 × 10^6^ M^−1^) for the CD units. This value results in a compact structure with a high steric hindrance limiting the rotational flexibility. The strength of the interaction was also illustrated in presence of adamantane-DOTA ligand with an association constant of 5.0 × 10^4^ M^−1^. This fitting provided an *r*_1_ value of only 10.8 mM^−1^ s^−1^, limited by a low hydration state, a slow water exchange rate, and a more flexible linker resulting in a faster local rotation. Competition experiments between HSA and ditopic chelate in presence of dendrimer showed that the paramagnetic probes were not displaced by HSA, the slight enhancement observed resulting from a variation of viscosity. Finally, the cleavage in presence of a reducing medium (TCEP) occurred within a few seconds and generated a drop of relaxivity of 23%, results confirmed by phantom experiments. As the metal complexes were not displaced by the protein, the authors described this supramacromolecular probe as a potentially suitable probe for applications *in vivo*.

**Fig. 20:**
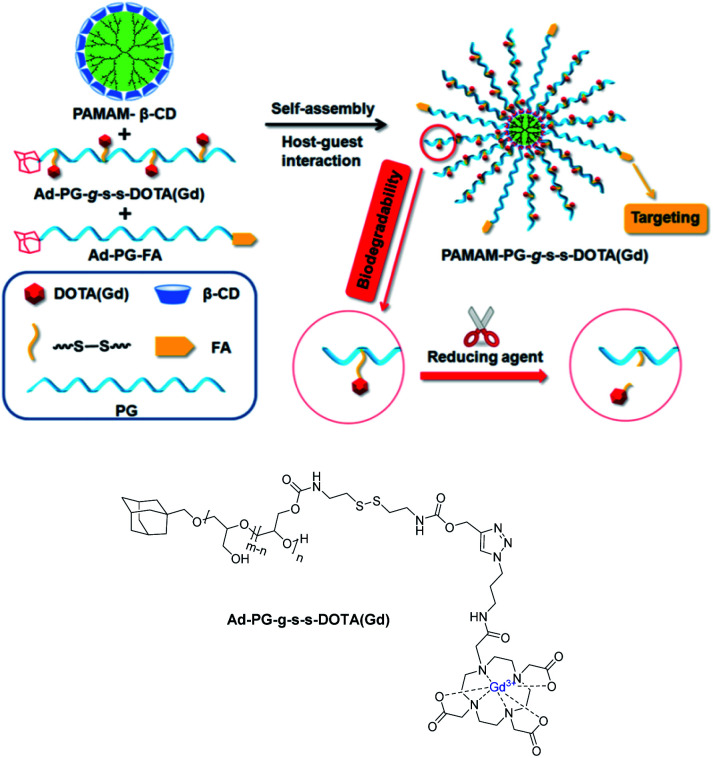
Illustration of the biodegradable nanoglobular and MRI CA. This figure has been adapted/reproduced from ref. 73 with permission from American Chemical Society, copyright 2018.

In 2018, Pei *et al.* designed a biodegradable targeted nanoglobular MRI contrast agent PAMAM-PG-g-s-s-DOTA(Gd) for tumor diagnosis (Fig. 20).^73^ The core of the nanostructure PAMAM-β-CD was constituted of β-cyclodextrin molecules immobilized onto the surface of poly(amidoamine) (PAMAM) dendrimer by an amidation reaction with 50% yield (16 β-CDs/dendrimer). Two guests were synthesized both based on adamantane bound to a poly(glycerol) PG spacer. The DOTA(Gd) chelate was firstly fixed *via* click chemistry to the modified biopolymer bearing sensitive disulfide bonds and the target molecule, folic acid (FA) was then fixed at the terminal site of PG. The larger size of the supramolecular assembly observed (around 3.95 to 7.52 nm) proved the host–guest recognition. The nanoparticle with both guests exhibited a higher relaxivity of 8.39 mM^−1^ s^−1^ at 0.5 T than in absence of FA-adamantane polymer guest; the hydrophilic PG promoting the access to water molecules. Good biocompatibility was observed based on absence of cytotoxicity and tissue toxicity. Indeed, the biodistribution study in main organs and tissues was performed after 7 days of injection and it has been shown that the highest level of gadolinium ion was detected in liver (0.25% ID per g). This value is much lower than the one obtained with macromolecular CA based on only PG moieties (3.8% ID per g). Brightest images were detected on cells of tumor-bearing mice with specific FA targeting. Finally, the redox cleavage of disulfide bonds using TCEP was validated releasing the small Gd chelate entities facilitating their quick clearance from the body. In *in vitro* conditions, after one hour, the relaxivity at the tumor location remained stable with a value of 4.80 mM^−1^ s^−1^ corresponding to the usual Gd(iii) chelate value. The signal was *in vivo* reduced after five hours proving the existence of degradability of disulfide bonds. This time is sufficient for MRI diagnosis and compatible with urinary excretion. Consequently, this smart MRI nanostructured contrast agent has a great potential for biomedical applications.

## 3. Cyclodextrins covalently linked to contrast agents

Cyclodextrins are known to be good carriers of contrast agents *via* covalent bonding (triazole, amide, ester) with the lower part of the cone (primary alcohols at 6-position). In the literature, many examples described the monofunctionalization or polyfunctionalization of CDs with various ligands such as commercial contrast agents such as DTPA, DOTA, carboxylate, or iminodiacetates ligands. Better relaxometric properties were obtained and many innovative applications in the field of biomedical imaging are now reported.

### 3.1 EDTA, DOTA, DTPA ligands

**Fig. 21:**
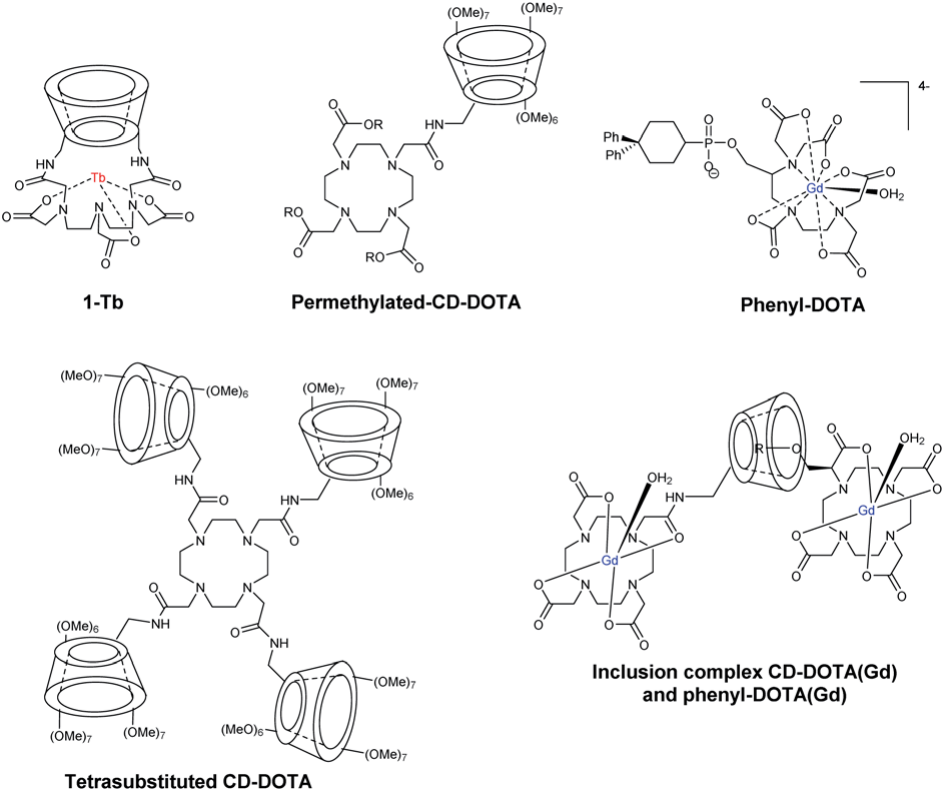
Structure of ligands, contrast agents and inclusion complexes.

Nocera in 1992 developed a new supramolecular architecture based on C6-monosubstituted β-cyclodextrin scaffold with aza crown ether ligand able to chelate metals.^74^ Firstly, they selected europium(iii) to get a photoactive tool with success. The luminescent properties remained identical in presence of CD. The formation of inclusion complex with benzene guest (5 × 10^−3^ M, *K*_a_ = 200 M^−1^) into the CD cavity led to an enhancement of a red emission due to an absorption energy transfer emission (AETE) process from the aromatic donor to the lanthanide acceptor. Later on, they optimized the initial conformation by fixing DTPA ligand by a double binding to the lower face of β-CD at A, D positions to chelate another lanthanide metal, the terbium(iii) (1-Tb, Fig. 21).^75^ DTPA ligand was then localized at a shorter distance from the cavity to optimize the energy transfer. Moreover, the carboxylate functions neutralized the terbium's positive charges which avoided all interferences. This first capped chemosensor was used to detect aromatic hydrocarbons. Indeed, the formation of inclusion complexes generated a brighter visible luminescence proving the molecular recognition.

Botta *et al.* in 2000 were the first who reported the synthesis of DOTA ligand covalently grafted on C6-position of more water-soluble permethylated β-CDs *via* amidation reaction (Fig. 21).^76^ Thus, mono and tetrasubstituted CD-DOTA chelates were obtained. Moreover, they reported monosubstituted DOTA *via* a phosphonate linker bearing two phenyl groups (*K* = 8 × 10^4^ M^−1^, Fig. 21). The impact of the formation of host–guest inclusion complex between CD-DOTA(Gd) and phenyl-DOTA(Gd) was studied by relaxivity measurements and a high relaxivity of 8.50 mM^−1^ s^−1^ (20 MHz, 298 K) was observed (Fig. 21).

**Fig. 22:**
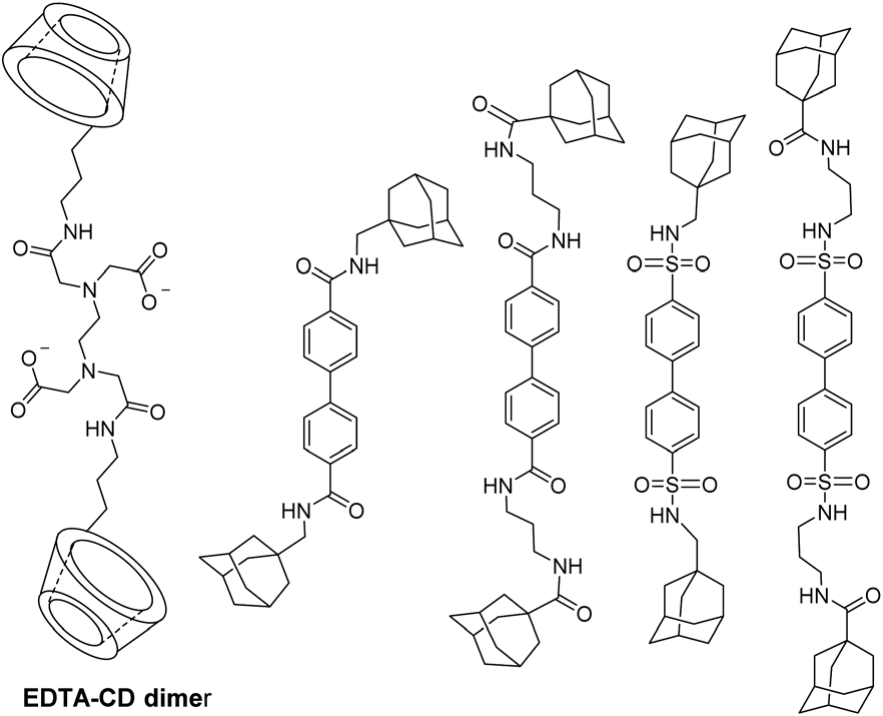
EDTA-CD_2_ dimer and bisadamantane guests.

EDTA bis-β-cyclodextrins (EDTA-CD_2_) ligand was also reported by Reinhoudt's team to study noncovalent binding with biphenyl adamantane derivatives which acted as antenna sensitizers for luminescence applications using Eu(iii) and Tb(iii) metals (Fig. 22).^77^^13^C NMR relaxation rate measurement was performed in presence of Gd(iii) and *p-tert*-butylbenzoate guest (*K*_a_ = 10^4^ M^−1^) and a complex with 1 : 2 (EDTA(Gd)-CD_2_ : guest) stoichiometry was observed.

**Fig. 23:**
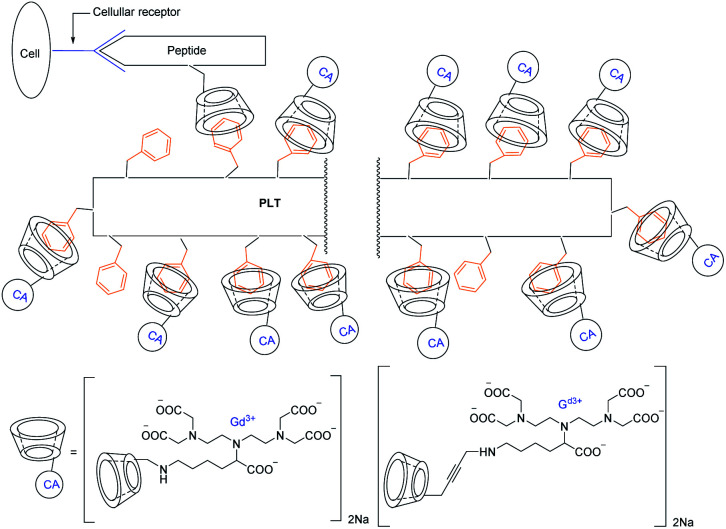
Schematic representation of a self-assembling MRI contrast agent.

Aime described three synthetic pathways to get three β-cyclodextrins covalently bound with amine function to DTPA (diethylentriaminopentaacetic acid) lysine ligand thanks to three different linkers (Fig. 23).^78^ They studied the molecular recognition with a polypeptide containing 53% tyrosine residues (PLT = poly-lysine-tyrosine) and an octapeptide vector (CCK8) bearing a β-CD. This peptide is specific to colecistokinine receptor present on the cell membrane. A stable and water-soluble supramolecular adduct was obtained. The nature of the linker did not make any change on the inclusion and the relaxometric properties. Indeed a relaxivity of 11.6 mM^−1^ s^−1^ (20 MHz, 25 °C) was measured for the three CDs-Gd(iii). However, the supramolecular interactions with the two peptides entailed a marked relaxivity with 32 mM^−1^ s^−1^ (20 MHz, 25 °C) due to a longer reorientational correlation time. The study of this phenomenon called proton relaxation enhancement (PRE) led to the affinity constant with PLT that was evaluated to *K*_a_ = 1.6 × 10^4^ M^−1^. This self-assembled model validated the capability to interact on a biological target site with a good MRI efficiency.

**Fig. 24:**
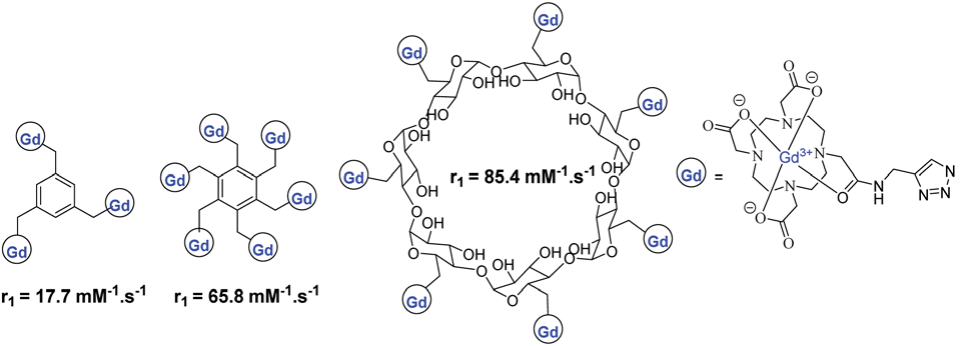
Structure of the MRI contrast agents with DOTA ligands and relaxivity values.

Meade grafted seven DOTA-Gd ligands on β-CD using click chemistry and compared the relaxometric properties with aromatic scaffold bearing three and six DOTA(Gd) contrast agents also both linked with triazole units (Fig. 24).^79^ Relaxivities *r*_1_ of 5.9, 10.9, and 12.2 mM^−1^ s^−1^ per Gd (1.4 T, 37 °C) were observed with the three models. The rigid nature of the linker and high molecular mass of CD-DOTA(Gd)_7_ led to a remarkable global relaxivity of 85.4 mM^−1^ s^−1^. MR phantoms experiments were carried out with the three contrast agents. As expected, brighter images appeared with CD scaffold, and similar results were observed after cell incubation. Preliminary viability tests showed promising biocompatibility for cell tracking imaging.

**Fig. 25:**
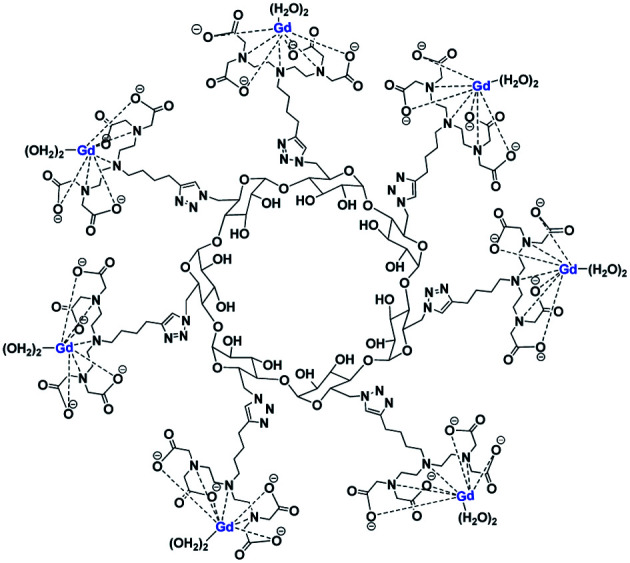
Structure of contrast agent Gd10 based on DTPA ligands and β-CD.

**Fig. 26:**
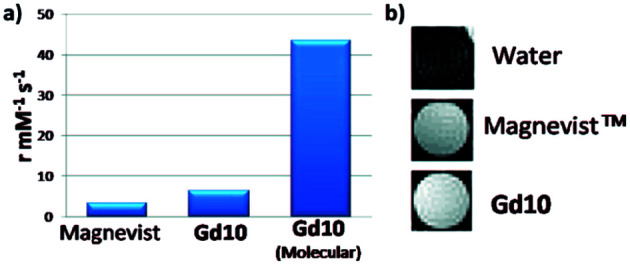
(a) Relaxivity of Magnevist and Gd10 at 9.4 T. (b) T1-weighted MR images of water, Magnevist and Gd10 at 4 T. This figure has been adapted/reproduced from ref. 80 with permission from American Chemical Society, copyright 2008.

The same year, Reineke reported a β-cyclodextrin decorated with seven DTPA(Gd) arms immobilized by click chemistry (Fig. 25).^80^ As the linker was fixed to the triazole through an amine function, a heptacoordinated Gd(iii) ligand was obtained with two water molecules present in its inner hydration sphere. Indeed, luminescence measurements were carried out in H_2_O and D_2_O on click cluster and an average of 1.8 water exchange sites was detected. A high relaxivity profile with 43.4 mM^−1^ s^−1^ corresponding to 6.2 mM^−1^ s^−1^ per Gd(iii) at 9.4 T, 37 °C was obtained corresponding to 94% increase by comparison with commercial Magnevist© (3.2 mM^−1^ s^−1^). A brightest signal and highest contrast were confirmed on the 4 T human MRI scanner (Fig. 26). Consequently, this paramagnetic macromolecular contrast agent can improve the diagnosis of disease thanks to this high MRI resolution.

### 3.2 Bimodal probes

**Fig. 27:**
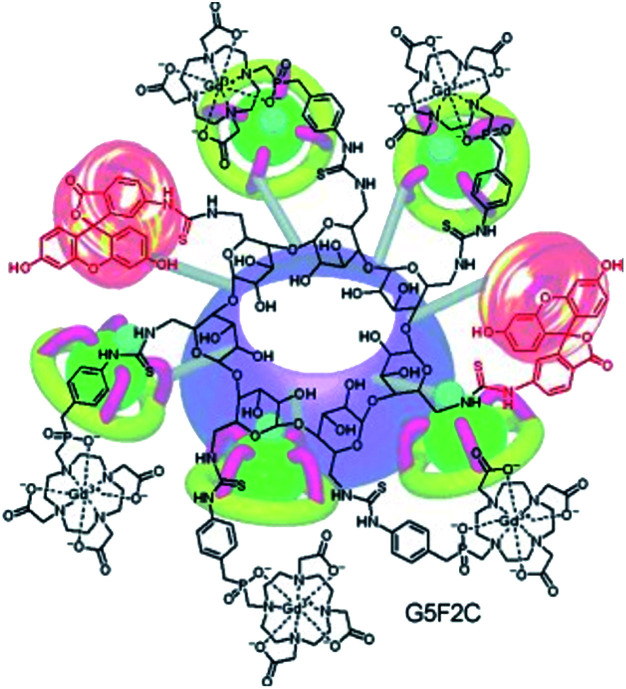
(GdL)_5_-F_2_-β-CD bimodal probe. This figure has been adapted/reproduced from ref. 81 with permission from John Wiley & Sons, Inc., copyright 2010.

In 2010, Kotek extended the concept by developing a bimodal probe based on the β-cyclodextrin scaffold with simultaneously MRI and fluorescence properties (Fig. 27).^81^ DOTA derivative chelates (DO3AP, L) were immobilized using thiourea bridges and an aminobenzyl phosphinic acid pendant leading to octacoordinated ligands (*q* = 1). Fluorescein tracers (F) were also grafted by isothiocyanate arm. A statistical composition of (GdL)_6.9_–F_0.1_–β-CD was obtained with a high relaxivity of 150 mM^−1^ s^−1^ or 22 mM^−1^ s^−1^ per Gd(iii) (0.5 T, 25 °C). The authors used this efficient probe to study *in vitro* pancreatic islets and rat mesenchymal stem cells. No toxicity and internalization into cells were observed by fluorescence and MRI phantom experiments. A significant highlighting was visualized even at low concentration (1 mM per Gd) and very low toxicity was detected in these conditions due to the low level of fluorescent molecule per CD. Finally, a quantitative fluorescent measurement proved that the contrast agent stayed in the intracellular space even after 24 hours allowing future *in vivo* imaging applications.

**Fig. 28:**
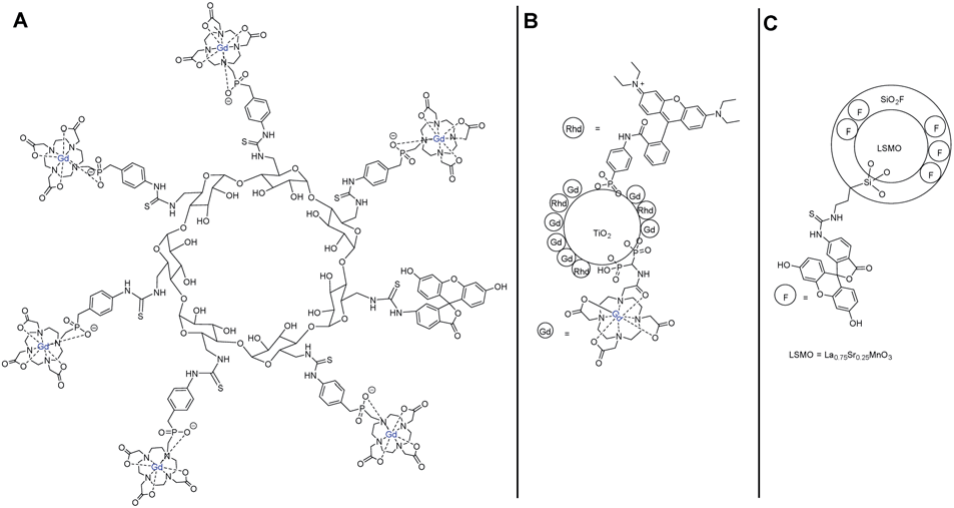
Structures of the bimodal contrast agents (CA) (A) β-CD (T1 CA), (B) TiO_2_ nanoparticle (T2 CA), (C) hydrid silica (T1 CA).

Then other macromolecules were synthesized using the same phosphonate DO3AP ligand and linkage without a fluorescent marker on α- and β-cyclodextrins.^82^ A slightly higher relaxivity value was detected with 21.6 and 21.0 mM^−1^ s^−1^ for α-CD and β-CD, respectively at 1.5 T. The authors carried out the work through a comparative study between three bimodal Gd(iii) and Mn(iii) probes applied to visualize pancreatic islets transplantation by optical and MRI imaging *in vitro*.^83^ The contrast agents (CAs) were carried by a β-CD, a TiO_2_ nanoparticle, and a hydrid silica (Fig. 28). The fluorescent properties were produced from fluorescein or rhodamine B molecules linked to the support. The MRI experiments indicated that the T2 contrast agent was a superior marker with a better contrast enhancement than T1 CAs *in vitro* and *in vivo* after 7.5 minutes of acquisition scans but with dark hypointense spots (negative contrast). Indeed at a higher magnetic field (4.7 T) and temperature (37 °C), the CD-Gd CA relaxivity dropped to 10 mM^−1^ s^−1^ increasing the acquisition time required (23 minutes). Similar results were obtained for the TiO_2_ nanoparticle. The three probes were detected inside the islet cells by fluorescence and induced no cell toxicity. This result underlined the great interest of dual detection systems to improve the monitoring of sensitive cells.

**Fig. 29:**
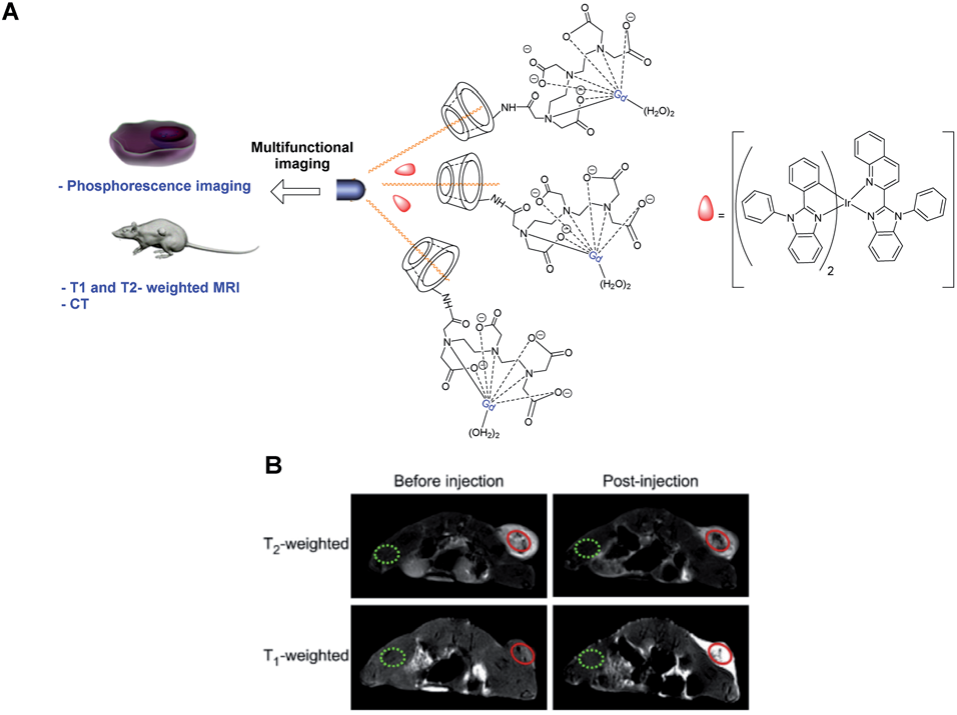
(A) Structure of the contrast agent and its application in multifunctional imaging. (B) T2 and T1-weighted MR images of the tumor obtained before and 30 min after intratumoral injection of DyNPs-Gd-Ir. This figure has been adapted/reproduced from ref. 84 with permission from Elsevier, copyright 2014.

Zhou and Liao designed in 2014 a T1- and T2-weighted MRI/CT/phosphorescence multimodal imaging agent using T2 contrast agent based on oleic acid-coated nanorods NaDyF_4_ (Fig. 29).^84^ β-Cyclodextrin was selected to form an inclusion complex with the oleic chain. T1 paramagnetic DOTA-Gd chelate was immobilized on the C6-position of the crown *via* amidation reaction. Moreover, the dysprosium inducing a high X-ray absorption, a computed tomography (CT) analysis was conducted. A phosphorescent probe, an iridium complex, was loaded within the surface of hydrophobic layers of the nanoparticle leading to a multifunctional imaging system. Thus, phosphorescent cell labeling, positive T1, negative T2-weighted MRI (3 T), and CT experiments could be performed. This multimodal imaging platform DyNPs-Gd-Ir was tested in HeLa cell lines *in vitro* and *in vivo* and exhibited 2 weeks water solubility, an intense yellow-red emission (600 nm), long phosphorescent lifetimes (0.52 ms), good X-ray attenuation (10 mg mL^−1^, HU = 158), and effective T1- and T2-weighted MRI enhancement (*r*_1_ = 4.65 mM^−1^ s^−1^, *r*_2_ = 7.68 mM^−1^ s^−1^) with low cytotoxicity (Fig. 29).

### 3.3 Acetate and iminoacetate ligands

In 2008, Delangle and Fries's team published a contrast agent based on per(3,6-anhydro)-α-cyclodextrin with six methylcarboxylate functions at 2-positions ready to chelate Gd(iii) ion.^85^ In this configuration, one and two lanthanides were localized closer to the CD cavity optimizing the interactions and exchanges with water molecules. Thus, a relaxivity of 22.4 mM^−1^ s^−1^ (400 MHz, 298 K) was observed. A low thermodynamic stability constants (log β = 7.5 and pGd = 8.9) were measured. To evaluate the transmetalation mechanism at the origin of the *in vivo* toxicity, a potentiometric study showed selective coordination in favor of Gd(iii) ion over Cu(ii), Zn(ii), and Ca(ii) ions due to a better fitting of the larger size of the lanthanide. Then, the contrast agent was used *in vivo* to quantify blood–brain volume in mice whose blood–brain barrier was damaged, without resulting in acute toxicity.^86^ The results showed confinement of gadolinium chelates in the vascular space unlike the DOTA(Gd) free which can enter into the tumor disrupting the measurement of the hemodynamic parameter. No nephrotoxicity or hemolysis was observed unlike with native cyclodextrins.

**Fig. 30:**
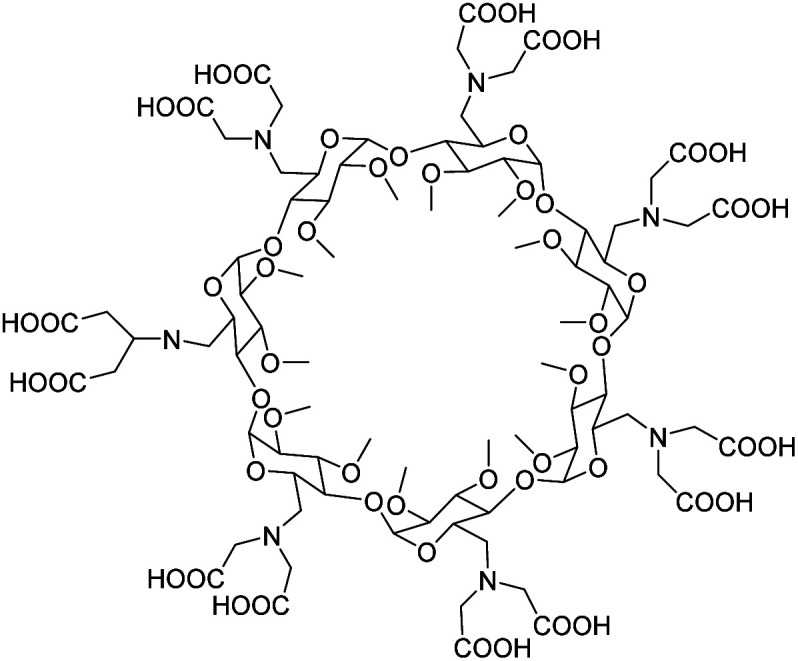
Structure per-6-bis(carboxymethyl)aminopermethylated β-CD.

Yannakopoulou *et al.* prepared in 2010 permethylated α-, β- and γ-CDs decorated with 6 to 8 functionalized bis(carboxymethyl)amino groups at the primary side of the macrocycle (Fig. 30).^87^ Semi-empirical quantum mechanical calculations, performed by the PM3 method, luminescence titrations, and mass spectrometry showed the formation of multimetal complexes. Coordinations of 2 to 4 metal ions (Eu(iii), Tb(iii), and Gd(iii)) occurred depending on the structures *via* 2 carboxylates and one amine of two adjacent ligands, and an oxygen atom of the glucopyranose of the macrocycle. Indeed, the luminescence lifetime of the complexes gave a number of water molecules coordinated around *q* = 1.5. It resulted in an enhancement in relaxivity of 4 to 10 times greater (8.8 mM^−1^ s^−1^) than with a commercial contrast agent with an increment of 9.3, 10.5, and 23.2 mM^−1^ s^−1^ per Gd for α-, β- and γ-CDs, respectively (100 MHz, 2.35 T, 25 °C). MTT tests using human skin fibroblasts did not show any toxicity. The luminescent properties were not modified after the formation of the inclusion complex with various aromatic moieties due to a too large guest-metal distance not enabling the energy transfer process. However, the stability of the complexes was sufficient to sustain high relaxivity values in human blood plasma over 2.5 hours.

**Fig. 31:**
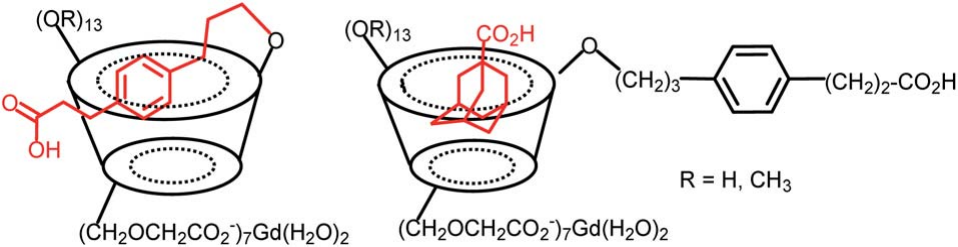
C6-Peracetate β-cyclodextrins contrast agents with intra and intermolecular inclusion complexes.

Gouhier's team synthesized C6-peracetate β-cyclodextrins used as a ligand to coordinate Gd(iii) ion (Fig. 31).^88^ Native and permethylated β-CD were tested to evaluate the influence of hydration sphere on relaxivity results. Both structures have similar stability constant *K*_LGd_ = 6.6, hydration number (*q* = 2–2.5), and molecular weight but provided different relaxivities values of 6.5 and 4.6 mM^−1^ s^−1^ (37 °C, 0.5 T) for native and permethylated β-CD, respectively. Consequently, hydroxyl groups on the crown of CD generated stronger affinity for water molecules thanks to hydrogen bonding underlying the nonnegligible participation of the second coordination sphere in the relaxometry effect. The efficiency of the gadolinium complexes obtained was successfully tested *in vitro* and *in vivo* in mice at a cardiac level.^89^ Brighter image was achieved in presence of native cyclodextrin compared to permethylated one confirming the importance of the functionalization of the macrocycle in the design of MRI contrast agent. The same team studied the effect of the formation of inclusion complex on the MRI signal on similar structures.^90^ They observed a significant increase in MRI signal (+58% and +21% for perhydroxylated and permethylated CDs) during the formation of inter- and intramolecular inclusion complexes using a flexible linker with hydrocynnamic acid and an adamantane acid molecules (Fig. 31). Under these conditions, the variation of the hydration spheres, the molecular weight, and the symmetry of the supramolecular complex disturbed the signal. This result makes feasible the visualization by MRI of the vectorization of encapsulated drugs opening the way to new theranostic strategies.

**Fig. 32:**
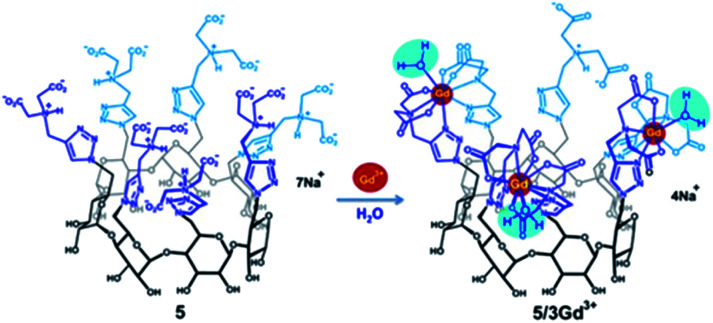
Formation of contrast agent based on iminodiacetate ligands with 3 Gd(iii).^91^

In 2018, Ling and Gouhier reported a β-cyclodextrin decorated with seven iminodiacetate ligands able to octacoordinate one to three Gd(iii) atoms (Fig. 32).^91^ To study the influence of the macrocycle an oligosaccharide bearing two ligands chelating one Gd(iii) lanthanide was synthesized as a model. Thus, the relaxometric studies revealed very high relaxivity values of 12.67 mM^−1^ s^−1^, 27.69 mM^−1^ s^−1^ at 37 °C, and 0.5 T (13.8 mM^−1^ s^−1^ per Gd), and 42.14 mM^−1^ s^−1^ (14.0 mM^−1^ s^−1^ per Gd) for 1 : 1, 2 : 1, and 3 : 1 Gd(iii) : CD ligands complexes corresponding to 2.7, 3.0, and 3.1 times the one obtained in presence of the maltoside model (4.61 mM^−1^ s^−1^). Potentiometry and kinetic stability studies have assessed the ligand/metal binding interactions and pGd indispensable values for biomedical applications in presence of other endogenic metal ions. Thus, higher stability (log βGd = 25.1 and pGd = 15.6 for mononuclear complex) was observed by comparison with DOTA(Gd) contrast agent (log βGd = 25.3 and pGd = 12.1) used as medical reference proving the strong potential of this new combination ligand/cyclodextrin. Moreover, the seventh functionalized flexible arm remaining is still available as a future linker for a biological target.

### 3.4 Polymers of cyclodextrins

**Fig. 33:**
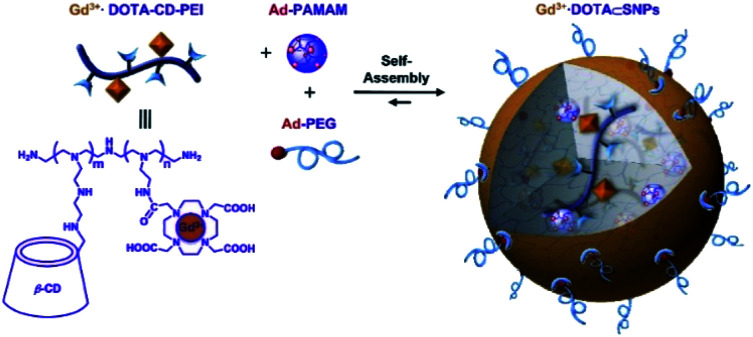
Schematic illustration of the preparation of Gd^3+^·DOTA-encapsulated supramolecular nanoparticles. This figure has been adapted/reproduced from ref. 92 with permission from Elsevier, copyright 2011.

**Fig. 34:**
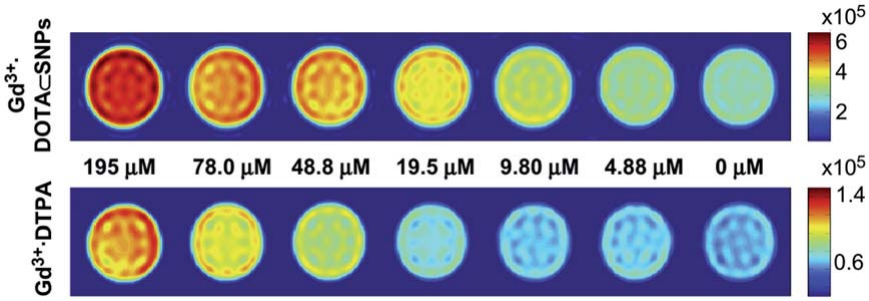
T1-weighted imaging results (7 T) of DTPA-Gd and nanoparticles from 0 μM to 195 μM. This figure has been adapted/reproduced from ref. 92 with permission from Elsevier, copyright 2011.

Tseng published in 2011 a supramolecular nanoparticle-based of host–guest inclusion complexes (Fig. 33).^92^ The host was represented by a β-cyclodextrin covalently conjugated onto a DOTA(Gd) chelate linked by a polyethylenimine (PEI). Two guests were used both with an adamantane group functionalized with a polyethylene glycol (PEG) or polyamidoamine hydrogel dendrimer (PAPAM). The various ratio of these building blocks were tested to optimize the molecular recognition process and to produce a remarkable relaxivity of 17.3 mM^−1^ s^−1^ (14 T, 295 K). Spherical morphology with an average diameter of 103 nm was obtained. This self-assembly provided paramagnetic properties used for *in vivo* imaging lymphatic drainage of mice. A brighter image 3.6 times than with DTPA appeared after injection (4.88 mM) proving the high sensitivity of the nanoparticle (Fig. 34). Sensitivity and relaxivity improvement were observed in presence of nanoparticles even at high dilution level. The signal intensity decreased to normal after 12 hours. The ICP-MS studies on dissected tissues were in good agreement with T1-weighted MRI results.

**Fig. 35:**
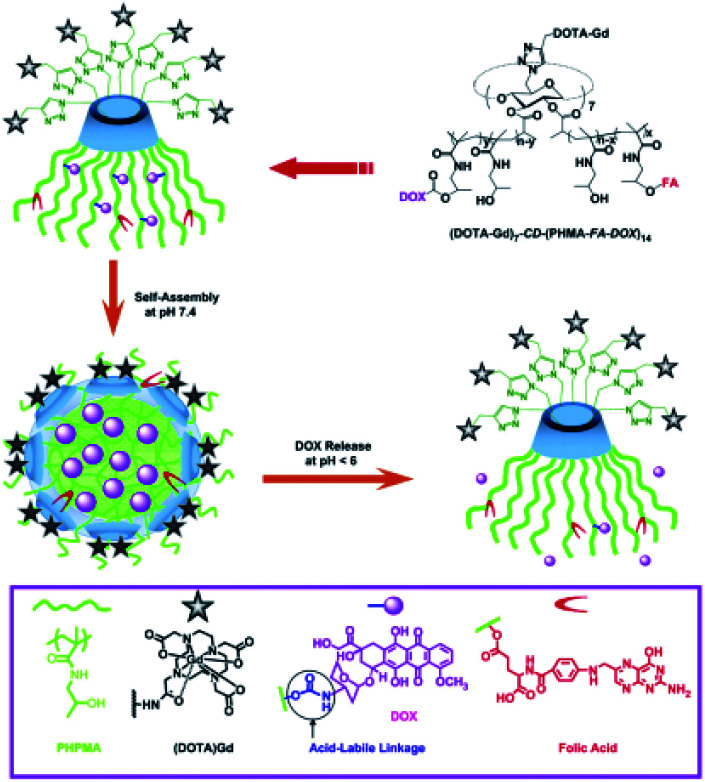
Fabrication of multifunctional micellar nanoparticles for theranostics based on star copolymers of β-CD, (DOTA-Gd)_7_-CD-(PHPMA-FA-DOX)_14_ covalently conjugated with doxorubicin fragments (DOX), folic acid (FA) and DOTA-Gd. The release of DOX is controlled by pH *via* the cleavage of the carbamate bonds between the DOX and HPMA arms. This figure has been adapted/reproduced from ref. 93 with permission from Elsevier, copyright 2012.

In 2012, Liu described multifunctional pH-disintegrable micellar nanoparticles (Fig. 35).^93^ Both sides of the β-cyclodextrin scaffold was used as support to immobilized on primary side 7 DOTA(Gd) moieties by click chemistry and on the secondary side 14 amphiphile poly(hexylmetacrylate) (PHMA) arms covalently anchored with doxorubicin (DOX) and folic acid (FA) *via* acid-labile carbamate linkages and ester bonds, respectively. The formation of self-assembly micellar nanoparticles occurred thanks to the hydrophobic core of DOX-PHMA conjugated arms and the high solvated DOTA(Gd) moieties forming the outer corona. After interaction of FA in cancer cells, the spherical nanoparticle (20–30 nm) delivers the drug on-site (12.6 DOX/star copolymer, 39.2 kDa) by hydrolysis of carbamate bonding under acidic microenvironment of tumor tissue leading to a 5 nm nanoparticle. *In vitro* a high relaxivity (*r*_1_ = 11.4 mM^−1^ s^−1^ at 1.5 T) and non-cytotoxic effect were observed up to concentration of 0.5 g L^−1^. The efficiency of the targeting was confirmed by the MRI visualization of the micelles accumulation in rats' liver and kidneys. The authors suggested an application of these pH-triggerable controlled release nanocarriers in the field of image-guided cancer chemotherapy.

**Fig. 36:**
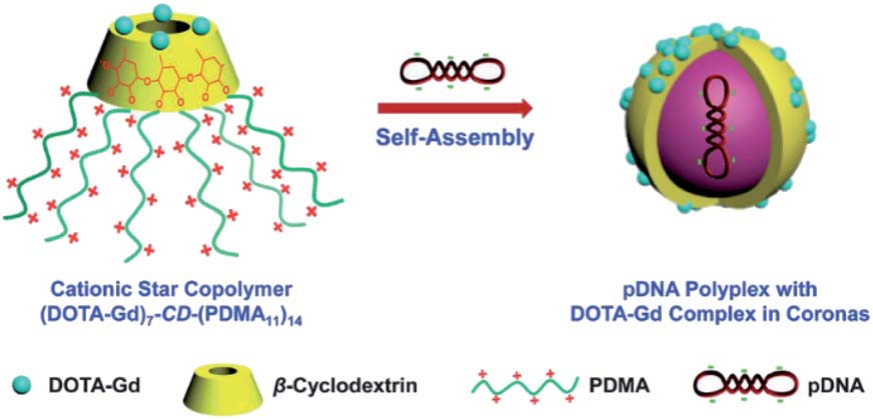
(DOTA-Gd)_7_-CD-(PDMA_11_)_14_ acting as pDNA delivery vectors and MR imaging contrast agents. This figure has been adapted/reproduced from ref. 94 with permission from Royal Society of Chemistry, copyright 2014.

Two years later, the same team designed a star copolymer based on β-cyclodextrin, DOTA(Gd), and polydimethylaminoethylmetacrylate (PDMA) to image by MRI pDNA polyplex (Fig. 36).^94^ In similar way seven paramagnetic chelates were linked to the lower face of the oligosaccharide using click chemistry. The upper face was persubstituted with cationic PDMA. Spherical nanoparticles with star-shaped chain topology of around 50 nm in diameter and high cell viability were observed. In presence of anionic plasmid DNA, a self-assembly occurred encapsulating the guest into the cationic lipophilic star *via* electrostatic interactions. This magnetic vector generated *in vitro* a high relaxivity of 10.9 mM^−1^ s^−1^ and reinforcement of the spot brightness in cells. The improvement in imaging signal could be due to synergistic effects: a restricted mobility of Gd(iii) chelate, an increase in the rotational correlation time, and a high local concentration of paramagnetic complexes. To conclude, this new supramolecular nanocarrier offers an interesting potential in the visualization of gene delivery theranostic applications.

A new theranostic nanoplatform able to release curmumin (CUR) encapsulated into a polymer of β-CDs (PCD) was reported.^95^ Simultaneously, T1 and T2 contrast agents were associated using DTPA-Gd and Fe_3_O_4_ nanoparticles both covalently immobilized on PCD, respectively. The efficiency of Fe_3_O_4_@PCD-DTPA-Gd showed no toxicity against normal and cancerous cell lines. *In vivo* MR imaging of the active CUR complex in mice exhibited both T1 positive and T2 negative dual contrast enhancements on the tumor cells and better therapeutic efficacy in cancer validating this theranostic probe.

### 3.5 Polyrotaxanes

**Fig. 37:**
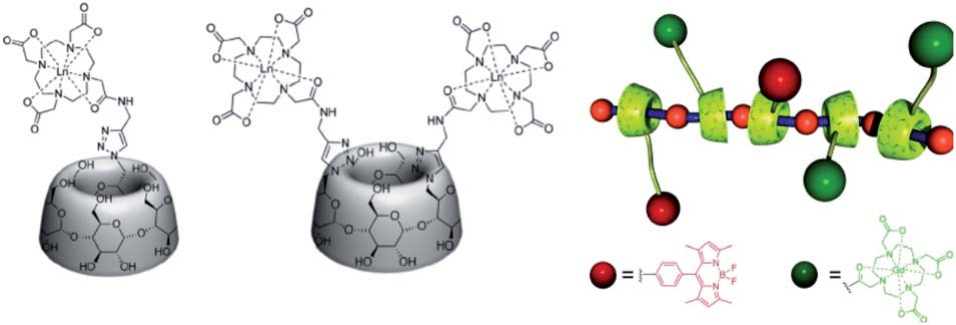
Structure of the contrast agents with one and two DOTA ligands. Polyrotaxane modelisation with CAs and Bodipy fluorescent tags. This figure has been adapted/reproduced from ref. 96 with permission from American Chemical Society, copyright 2014.

In 2014, the first MRI/fluorescent polyrotaxanes based on symmetrical β-cyclodextrins were described by Hasenknopf *et al.* (Fig. 37).^96^ The lower face of the macrocycle was substituted with one or two DOTA(Gd) chelates at A, D-positions using the efficient click chemistry. Relaxivities of 7.06 and 8.57 mM^−1^ s^−1^ were observed in presence of one and two contrast agents, respectively (37 °C, 0.5 T). PEG, poly(iminium), and poly(ammonium) chains were tested as axles of the pseudorotaxanes. However, only the alkylammonium chain provided sufficient affinity (log *K* = 3.0–3.4, 2.8 mM) with the modified CDs cavities and led to the supramolecular assembly with 55–69% of inclusion with 1 : 1 stoichiometry. A lower association constant was observed in presence of two bulkier DOTA ligands. An additional fluorescent tag based on Bodipy moiety linked to another β-CD by click chemistry was thread through the axle and the optic properties remained constant. Various ratios of molecular bricks were tested and high relaxivities between 17.43 to 20.95 mM^−1^ s^−1^ (20 MHz, 37 °C) were observed for the water-soluble polyrotaxanes corresponding to five-fold the value obtained with the commercial DOTA(Gd).

**Fig. 38:**
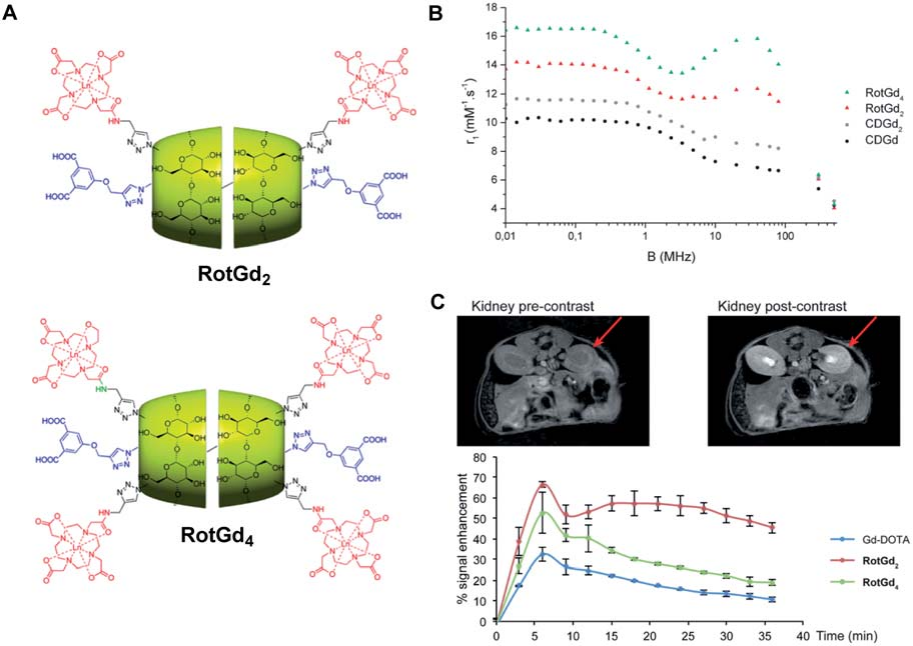
(A) Structure of the rotaxanes with 2 (RotGd2) and 4 (RotGd4) DOTA-Gd ligands. (B) ^1^H NMRD profiles of aqueous solution of CD-Gd, CD-Gd2, RotGd2, and RotGd4 at 37 °C. (C) MRI cross sections of the kidney pre and post injection (right, 40 min) of RotGd2. Dynamic MRI signal enhancement in renal cortex after CA injection. This figure has been adapted/reproduced from ref. 97 with permission from John Wiley & Sons, Inc., copyright 2017.

In 2017, this concept was extended to new supramolecular entities obtained in one-pot strategy and involving two cyclodextrins bearing one or two contrast agents and a small thread based on C12 alkyl chain stoppered to the ends by dicarboxylic acids function (Fig. 38).^97^ This head-to-head polyrotaxanes produced higher relaxometric properties with 12.30 and 15.70 mM^−1^ s^−1^ (20 MHz), for one or two DOTA(Gd) ligand/CD, respectively. The NMRD profile at 37 °C showed a hump characteristic of the rotational dynamics of macromolecular contrast agents such as polymers or liposomes. Moreover, the increase of relaxivity observed at higher temperature confirmed the structural rigidity proving that the relaxivity is limited by the water-exchange rate of the complex and not by its rotation. The biodistribution was studied *in vivo* on mice using 7 T MRI at 50 μmol kg^−1^ and the brightest image was visible 21 minutes after injection in the liver and kidneys. The main retention in kidneys and an absence of toxicity were observed. A slow elimination time (after 40 minutes) opens the perspective of biomedical applications. Consequently, the authors inferred that the control of such modular supramolecular entities could be a great bimodal platform for imaging.

**Fig. 39:**
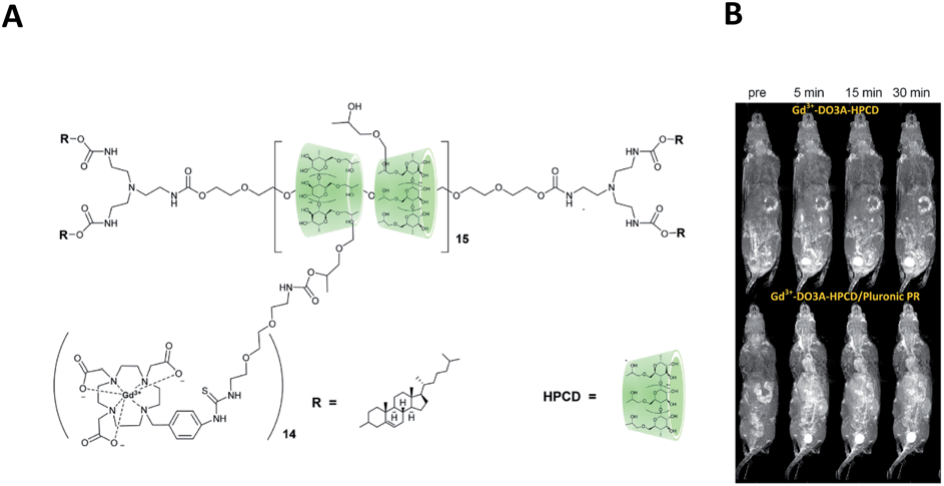
(A) Structure of the polyrotaxane based on DO3A ligands and pluronic axis. (B) 3D maximum intensity projection images (T1-weighted) of Balb/c mice injected with Gd^3+^-DO3A-HPCD (top row) or Gd^3+^-DO3A-HPCD/Pluronic PR (bottom row) at a 0.03 mM Gd per kg dose after injection of contrast agent. This figure has been adapted/reproduced from ref. 98 with permission from American Chemical Society, copyright 2015.

Thompson's team reported a polyvalent polyrotaxane built up from noncovalent self-assembly composed of pluronic F127 and hydroxypropyl-β-cyclodextrin (HPCD) to enhance the visualization of vascular blood flow in MRI using DOTA(Gd) contrast agent (Fig. 39).^98,99^ Pluronic axle was composed of the poly(propylene oxide) block with cholesterol bulky endcaps. The paramagnetic chelate was fixed to the oligosaccharide through a flexible isothiocyanatobenzyl linker. The authors showed that a longer retention time (30 minutes) in mice was possible limiting the clearance through kidneys and improving the analysis conditions. Indeed, the monomer was cleared and accumulated in the kidneys and bladder after 5 minutes. Moreover, the MRI contrast property with the rotaxanated Gd(iii) was enhanced by comparison with the monomeric CD-DOTA(Gd) alone (7.82 mM^−1^ s^−1^). Thus, a strong relaxivity of 23.83 mM^−1^ s^−1^ m at 37 °C and 1.5 T was detected. This 3 fold improvement is assigned to the rigidity, the reduced rotation motion of the structure, and the hydrogen-bonded of cyclodextrin units. The biodegradability of the macromolecule caused no toxic effects. Consequently, this biocompatible MRI probe can be useful for cardiovascular imaging. The authors carried out the study to characterize the pharmacokinetic profiles, toxicities, and protein corona biodistribution, circulation time, and excretion of the polyrotaxanes by varying the ratio of the triblocks *in vivo*. Thus, the structures and dynamics of their rodlike morphologies influenced the biological interactions. Indeed, high threaded macromolecules were longer retained and were detected in the mice liver, whereas the lower percentage of cyclodextrin favored lung deposition and rapid clearance. Furthermore, the various architectures underwent no difference in toxicity or organs damage. However, the accumulation of lipoproteins in human serum around the polyrotaxane was reduced in presence of a high number of CDs. Indeed longer naked PEG axle promoted the surface receptors binding thanks to more accessible cholesterol end-caps. Hence, these findings improved the design of new MRI contrast agents for specific biological target tissues.

**Fig. 40:**
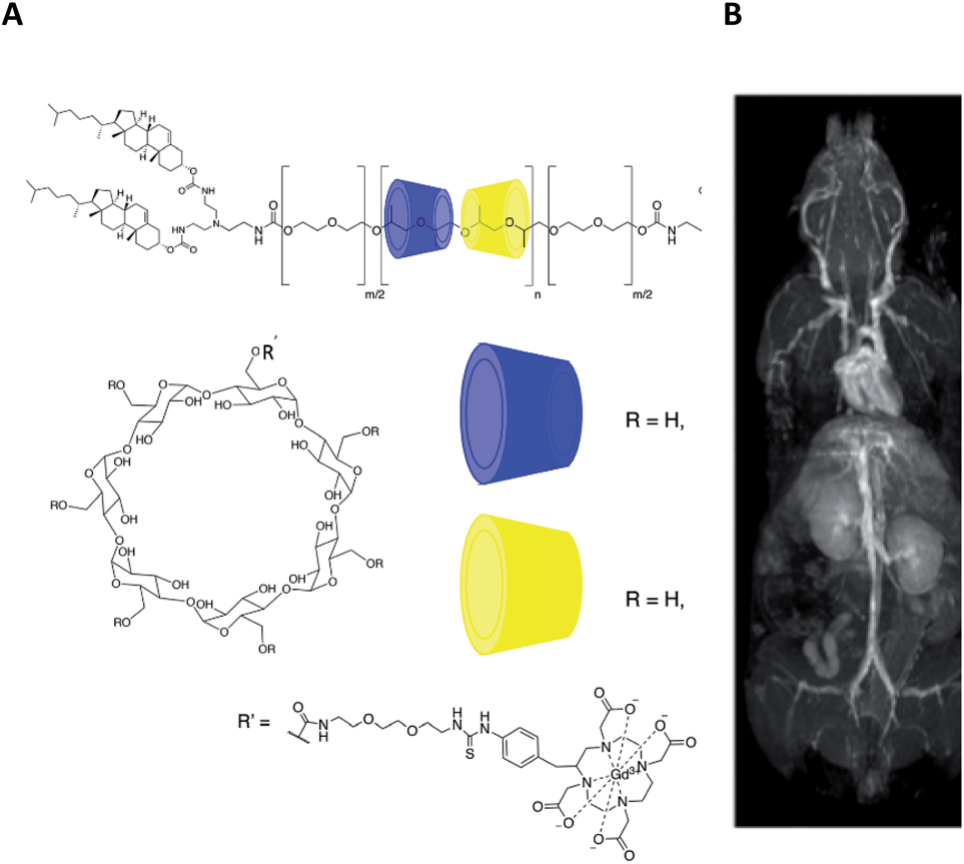
(A) Structure of Gd:DOTA-HPβCD/SBEβCD pluronic-based polyrotaxane contrast agents with cholesteryl end-caps. (B) 3D Maximum intensity projections of Balb/c mice recorded at 7 T after tail vein injection of 0.05 mmol Gd per kg bodyweight at 10 min. This figure has been adapted/reproduced from ref. 100 with permission from American Chemical Society, copyright 2018.

In 2018, Thompson and his group carried on the study of properties of β-cyclodextrins polyrotaxanes for *in vivo* MRI imaging (Fig. 40).^100^ They used two oligosaccharides substituted at six 6-positions with 2-hydroxypropyl (HPβCD) and 4-sulfobutyl groups (SBEβCD), respectively. In both cases, the seventh position supported a flexible linker with DOTA(Gd) chelate. Same pluronic thread and cholesteryl end-caps as previously described were used in various ratios of blocks polymers with functionalized CDs (40–50 mol% SBEβCD) to form mix polyrotaxanes with the amount of Gd varying from 4.5% to 11%, with molecular weights varying from 29.5 kD to 54.7 kD, and with sizes ranging between 110 and 230 nm. The large presence of negative sulfonate groups improved the water solubility of the assembly by limiting the aggregation phenomena.

Various T1-weighted MR image intensities were obtained with *r*_1_ from 7.9 to 22 mM^−1^ s^−1^ (25 °C, 7 T), the higher threaded structures showing the best enhancement. Long PEG blocks improved the water molecules exchanges with the Gd chelate and conferred a rod-like shape limiting the rotation and translation of the β-CD-DOTA(Gd) units around the polymer axle lowering the molecular tumbling rate. *In vivo*, an excellent contrast image was observed after 5–10 minutes in the heart, liver, and kidneys. A longer circulation time (1 hour) in the blood pool was analyzed after injection of 0.05 mmol Gd per kg in mice by comparison with commercial DOTAREM (20 minutes). The elimination of the contrast agent *via* renal of biliary routes was related to the design of the assembly. Here once again, a longer structure generated higher liver uptake. After serum exposure, the proteomic analysis showed 40% of interaction between polyrotaxane and lipoprotein corona due to interaction with the biantennary cholesterol end-caps.

However, the couple HPβCD/SBEβCD used generated less binding with protein corona than the native β-cyclodextrin due to electrostatic repulsion and/or limited hydrophobic interactions. The authors suggested that the rod-like structure was a good design to visualize blood circulation.

**Fig. 41:**
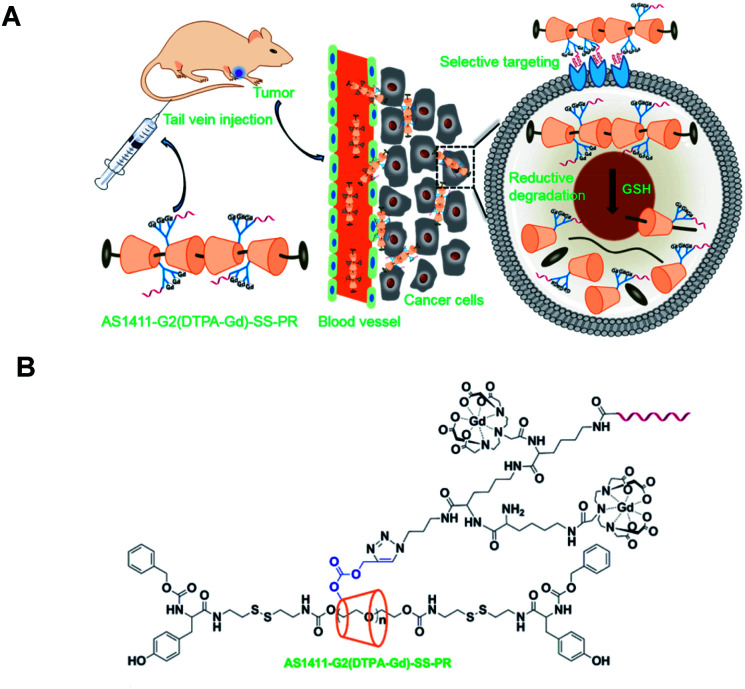
(A) Tumor-targeting and the reductive biodegradability of the biocleavable AS1411-conjugated-α-CD polyrotaxane-based MR contrast agent (AS1411-G2(DTPA-Gd)-SS-PR). (B) Structure of the contrast agent. This figure has been adapted/reproduced from ref. 101 with permission from American Chemical Society, copyright 2019.

In 2019, Liu and Pei described the synthesis of a biocleavable α-cyclodextrin polyrotaxane-based DTPA(Gd) contrast agent for targeted cancer cells (Fig. 41).^101^ The synthesis started by the host–guest complexation of the macrocycle and a poly(ethylene glycol) (PEG) chain containing disulfide linkages at both terminals. After functionalization of the two endpoints with bulky tyrosine groups, the alkynyl function was immobilized on C6-position of α-CD to allow click reaction with lysine azide derivative. The number of CDs threaded onto the PEG axle was calculated to be 23 by ^1^H NMR and the channel-type structure was confirmed using XRD analysis. From primary amino groups, DTPA ligands were grafted *via* amidation reaction and Gd(iii) was introduced leading to a new CA. The targeted molecule selected was the 26-mer guanosine which was coupled to a residual amino group of lysine dendron. This rich oligonucleotide aptamer is known to have a high binding affinity to nucleolin a multifunctionalized protein overexpressed on the surface of breast cancer cells forming a G-quadruplex structure. A brighter MRI image was observed due to a slowing down of the molecular rotation of the semirigid structure of the polyrotaxane and local concentration of Gd. The longitudinal relaxivity of the polyrotaxane was measured at 11.7 mM^−1^ s^−1^ that is higher than the DTPA(Gd) (4.16 mM^−1^ s^−1^ at 0.5 T). In presence of reducing intracellular glutathione (GSH) disulfide bonds were cleaved and the polyrotaxane slowly dethreaded decreasing *r*_1_ to 8.1 mM^−1^ s^−1^ after two hours of incubation. Cytotoxicity and histological experiments showed that the polyrotaxane was biocompatible and was cleared renally without long-term accumulation toxicity. *In vitro* and *in vivo* studies showed stronger interaction with the tumor region leading to higher accumulation and longer contrast imaging time.

### 3.6 Dendrimers

**Fig. 42:**
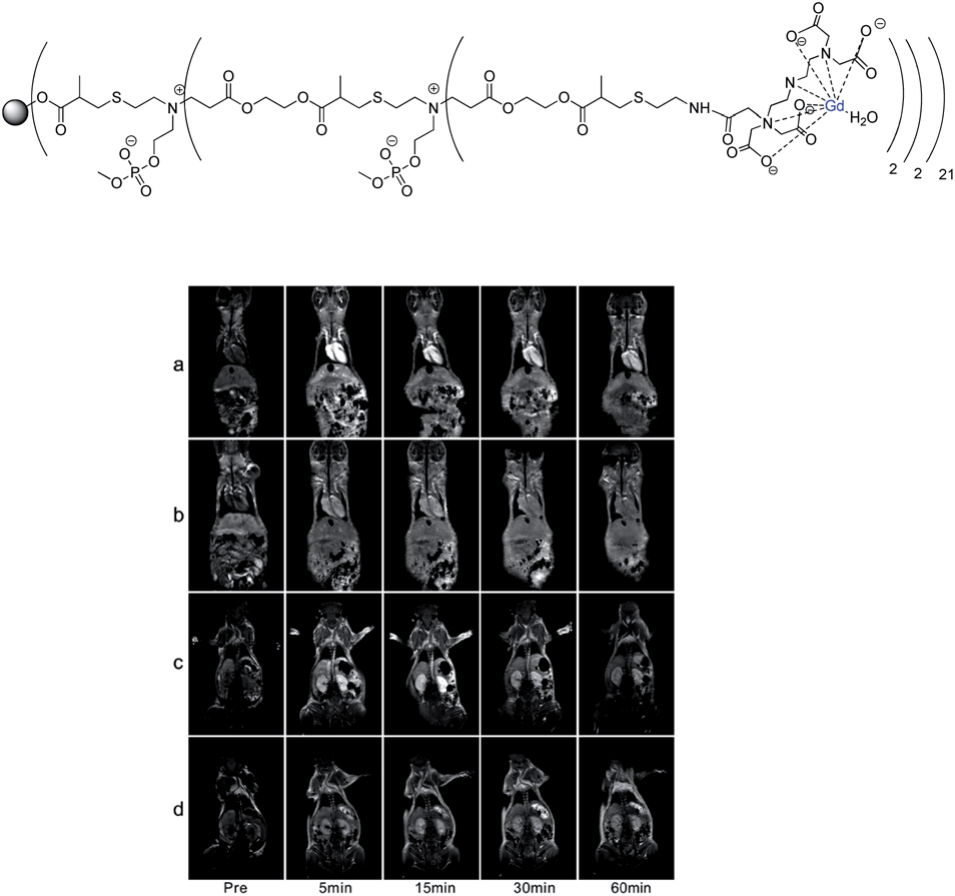
Structure of dendrimer. T1 weighted images of the mice injected with dendrimer (a and c) and Magnevist (b and d) at a dose of 0.1 mmol Gd per kg at the layers with heart (a and b) and kidneys (c and d). This figure has been adapted/reproduced from ref. 102 with permission from Royal Society of Chemistry, copyright 2012.

In 2012, Tang and collaborators reported biodegradable dendritic gadolinium contrast agents based on per-polyester substituted CD making a possible new biomedical application (Fig. 42).^102^ The dendritic contrast agent was easily obtained in three steps from methacrylated β-CD. In one pot, additional chains were substituted using cysteamine and 2-[(methacryloyl)oxy]ethylacrylate (MAEA) treatment. The synthesis ends with the quantitative immobilization of DTPA ligands on terminal amino groups and the coordination of GdCl_3_. Thus, three generations of dendritic structures were isolated (11.5, 29.3, 64.9 kDa). To improve the water solubility, dioxaphospholane-2-oxide (MOP) was added on tertiary amines leading to zwitter ionized dendrimers. These nanoparticles (2.8 to 8.6 nm) revealed a relaxivity around 11.7 mM^−1^ s^−1^ (0.5 T, 32 °C), which is 2.7 times that of a clinically used small-molecule CA (Magnevist®).

Similar longitudinal relaxation rates were detected for all generations of dendrimers due to the internal flexibility of the linkers in the assemblies. *In vitro*, fast hydrolysis (50%, 6 hours) in presence of esterase which is abundant in the cytoplasm was observed. *In vivo*, compared with commercial CA, the dendrimer showed longer blood retention (60 minutes) and similar retention in mice tissues proving its biodegradability property.

**Fig. 43:**
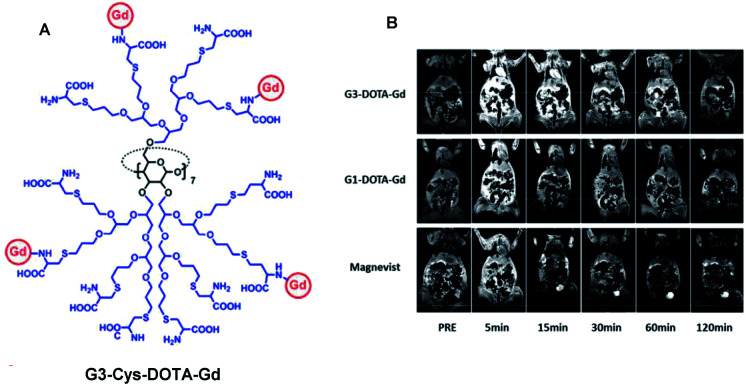
(A) Structure of contrast agent G3-Cys-DOTA-GD. (B) T1-weighted images after injection of contrast agent in the hearts. This figure has been adapted/reproduced from ref. 103 with permission from Royal Society of Chemistry, copyright 2016.

In 2016, Tang and coworkers used a β-cyclodextrin core as support of dendrimers functionalized with MRI DOTA(Gd) chelates (Fig. 43).^103^ The dendrimer was composed of zwitterionic polyglycerol linked to the oligosaccharide at 2,3 and 6-positions. l-Cysteine was introduced into the glycol chain as the anchor site of the paramagnetic ligand. Partial conjugation was obtained and 6.3% of Gd/dendrimer was measured. Even at this low concentration, a high contrast enhancement of 14.3 mM^−1^ s^−1^ was observed with this extracellular dendritic contrast agent in blood pools. This property was confirmed after injection into mice (0.1 mmol Gd per g) that produced a stronger signal than with commercial DOTA(Gd) such as 1.4 times after 5 minutes. A slower decay rate after 15 minutes was observed and elongation of the MRI time window up to 2 hours. The hyperbranched structure showed an anti-fouling property against protein adhesion in the liver. No cytotoxicity and cellular uptake were observed by ICP-MS after 10 days even at a high concentration of up to 1 mg mL^−1^ of injection into mice in contrast to smaller CA or polyaminoacide PAMMA-CA dendrimers. The absence of interactions with the mononuclear phagocyte system provides access to a promising imaging probe delivery.

**Fig. 44:**
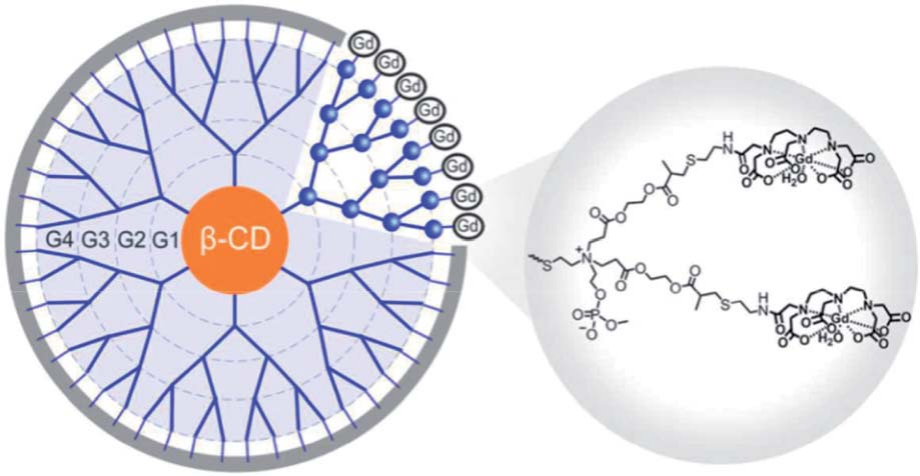
Structure of the dendrimer contrast agent. This figure has been adapted/reproduced from ref. 104 with permission from Royal Society of Chemistry, copyright 2017.

In 2017, the same team decorated a β-CD with a dendrimer functionalized with DTPA(Gd) chelates to better image liver metastases (Fig. 44).^104^ Indeed, the diagnosis of such a tumor is challenging because the current contrast agents accumulate in hepatocytes and Kupffer cells instead of cancer cells. The DTPA ligands were introduced on the external amino groups and a zwitterionic form was obtained by addition of phosphate function. Better retention in liver cancer cells was observed due to higher permeability through metastasized lesions. Because of the rigidity of the dendritic spherical structure (9 nm, 337 kDa) an enhancement of relaxivity (15.7 mM^−1^ s^−1^ at 0.5 T and 32 °C) was obtained. These ester bonds were hydrolyzed into small fragments (50% in 4 hours incubation, pH = 7.4) leading to a biodegradable nanoparticle. All these properties were confirmed *in vivo* experiments with the visualization of brighter images and good renal excretion. This supramolecular structure can be tailored for personalized treatments.

**Fig. 45:**
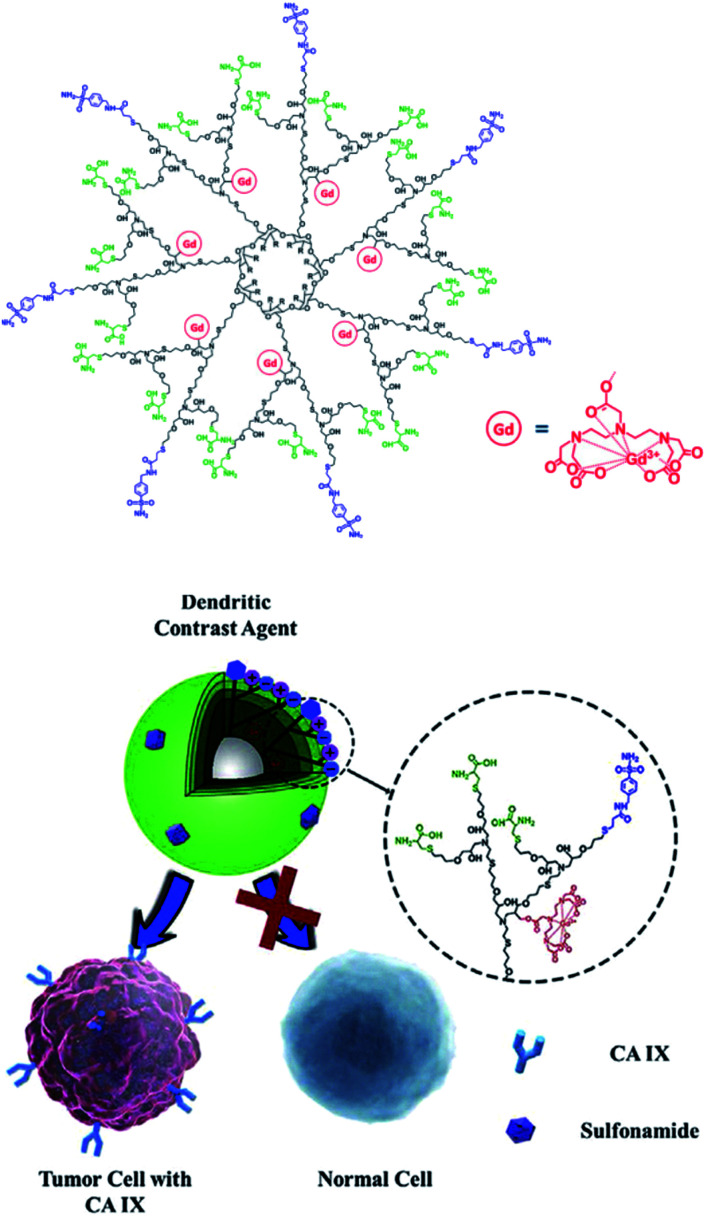
Structure of the hypoxia-targeting dendritic MRI contrast agent. This figure has been adapted/reproduced from ref. 105 with permission from Elsevier, copyright 2019.

These researchers designed in 2019 the structure of a dendrimer to target hypoxia imaging of solid tumors (Fig. 45).^105^ 6*O*-Per-dendrimer-β-CD was synthesized using efficient and simple epoxy-amine and thiol–ene reactions from 6*O*-perallyl-β-CD. DTPA ligands were immobilized by esterification reaction on internal secondary hydroxyl groups and dendrimer-contrast agent was obtained after addition of GdCl_3_. Sulfonamide was selected as a hypoxia-targeting group and a zwitterion (cysteine) used to avert unspecific protein adhesion and prolong the circulation time. Both functions were added to the residual external unsaturated chains. ^1^H NMR study indicated that *ca.* 30 DTPA ligands and 10 targeting groups were conjugated onto each dendrimer. Hydrodynamic diameter of 6.2 nm was obtained for this spherical 3D structure. The rigidity of the dendrimer increasing the rotational correlation time, a relaxivity value up to 11.6 mM^−1^ s^−1^ per Gd (0.5 T, 32 °C) was evaluated that is 2.7 higher than Magnevist®. The signal which disappeared after 30 minutes after the injection of DTPA(Gd) was still strong after 4 hours with the dendrimer-DTPA(Gd). Cellular uptake was confirmed by fluorescence after labeling of the structure using rhodamine B and Cy5.5. *In vitro* and *in vivo* MRI experiments (3 T) on orthotopic breast tumors in a mouse model showed a strong affinity for hypoxic cancer cells with higher cellular uptake and better accumulation (60.1%) in presence of sulfonamide group by comparison with cysteine groups (18.9%). Similar retention in all organs and tissues was observed compared with DTPA(Gd) validating the hypoxia-targeted contrast agent efficiency.

## 4. Conclusions and outlook

The first MRI contrast agent appeared in 1988 to improve the contrast of the image, the accuracy of the diagnosis, and consequently the survival of patients. From then a pioneering work mainly managed by an Italian group based on the use of cyclodextrin scaffold led to fundamental results allowing us to better understand the mechanism and the interactions which could influence positively the efficiency of the paramagnetic chelates. Two strategies have been reported, one uses the hydrophobic property of the internal cavity of the macrocycle using noncovalent host–guest inclusion complex with a modified contrast agent, the second uses the oligosaccharide as support by immobilizing covalently the contrast agent. In both approaches, cyclodextrins have a positive impact on MRI signal mainly due to an enhancement of the rotational correlation time.

DOTA, DTPA contrast agents were functionalized with aromatic and aliphatic groups to favor the inclusion into the cavities of cyclodextrins, dimer, trimer, and polymer of cyclodextrins. This concept validated, it was applied to biomedical applications using nanoparticles, bimodal probes using Gd(iii) and ^19^F or near-infrared imaging and, nanocapsules sensitive to reducing environment. *In vivo* tests showed that high Gd(iii) loading and high water molecules density of the macromolecular assemblies led to better MRI properties. These biocompatible macrostructures led to weak hepatic accumulation, good excretion by renal filtration, possible intravenous administration, and internalization into tumor cells.

Cyclodextrins were covalently linked to contrast agents based on EDTA, DOTA, DTPA, acetate, and iminoacetate ligands. Fluorescent and paramagnetic bimodal probes have been described. Polymers of cyclodextrins, polyrotaxanes, and dendrimers were associated with contrast agents with success.

To conclude in order to better design MRI contrast agents, cyclodextrins used as support or hosts were modulated for *in vitro* and *in vivo* biomedical applications. Their high molecular weights increase significantly the relaxivity values that can be reinforced by the presence of hydrophilic medium and high Gd(iii) loading.

Through many biological examples, it was observed longer residence time, better clearance, and absence of toxicity. Smart contrast agents have been reported able to be modified in presence of biological redox medium perturbing the MRI signal. The validation of bimodal probes and the possible modification of the imaging signal should open the way to new medicinal development in the future to visualize the efficiency of the treatment and improve the access to personalized medicine. The strategies developed seem to be promising for the *in vivo* targeted delivery of Gd(iii) complexes. Indeed, the recognition of specific biomarkers generated sufficient signal enhancement for detection.

Surprisingly, the use of these supramolecular complexes has not been described at higher clinical phases and will require high industrial investment. Easy, low-cost, and efficient click chemistry described to immobilize the paramagnetic chelates could afford a scale-up necessary to start clinical application. Consequently, cyclodextrins have a great potential for the design of a new generation of MRI contrast agents as drug delivery vectors and support for diagnosis. This dual property is an attractive tool that can open the way to the challenging development of new theranostic MRI probes in the forthcoming years.

## Funding

This work was supported by the Minister of Research and Technology (MRT) (PhD of B. S. S. B.). This work has been partially supported by University of Rouen Normandy, INSA Rouen Normandy, the Centre National de la Recherche Scientifique (CNRS), European Regional Development Fund (ERDF), Labex SynOrg (ANR-11-LABX-0029), Carnot Institute I2C, the graduate school for research XL-Chem (ANR-18-EURE-0020 XL CHEM), and by Région Normandie.

## Author contributions

GG writing-original draft, manuscript concept and design, funding acquisition, manuscript revision/review/editing. F. E. manuscript revision/review. B. S. S. B. manuscript figures, tables and revision. All authors contributed to the article and approved the submitted version.

## Conflicts of interest

The authors declare no conflict of interest.

## Supplementary Material
